# Highly Efficient Generation of Canker-Resistant Sweet Orange Enabled by an Improved CRISPR/Cas9 System

**DOI:** 10.3389/fpls.2021.769907

**Published:** 2022-01-11

**Authors:** Xiaoen Huang, Yuanchun Wang, Nian Wang

**Affiliations:** Department of Microbiology and Cell Science, Citrus Research and Education Center, Institute of Food and Agricultural Sciences, University of Florida, Lake Alfred, FL, United States

**Keywords:** CRISPR/Cas9, citrus, canker, HLB, *CsLOB1*, susceptibility gene, EBE, transcription activator-like effector

## Abstract

Sweet orange (*Citrus sinensis*) is the most economically important species for the citrus industry. However, it is susceptible to many diseases including citrus bacterial canker caused by *Xanthomonas citri* subsp. *citri* (*Xcc*) that triggers devastating effects on citrus production. Conventional breeding has not met the challenge to improve disease resistance of sweet orange due to the long juvenility and other limitations. CRISPR-mediated genome editing has shown promising potentials for genetic improvements of plants. Generation of biallelic/homozygous mutants remains difficult for sweet orange due to low transformation rate, existence of heterozygous alleles for target genes, and low biallelic editing efficacy using the CRISPR technology. Here, we report improvements in the CRISPR/Cas9 system for citrus gene editing. Based on the improvements we made previously [dicot codon optimized Cas9, tRNA for multiplexing, a modified sgRNA scaffold with high efficiency, citrus U6 (CsU6) to drive sgRNA expression], we further improved our CRISPR/Cas9 system by choosing superior promoters [*Cestrum yellow leaf curling virus* (CmYLCV) or *Citrus sinensis* ubiquitin (CsUbi) promoter] to drive Cas9 and optimizing culture temperature. This system was able to generate a biallelic mutation rate of up to 89% for Carrizo citrange and 79% for Hamlin sweet orange. Consequently, this system was used to generate canker-resistant Hamlin sweet orange by mutating the effector binding element (EBE) of canker susceptibility gene *CsLOB1*, which is required for causing canker symptoms by *Xcc*. Six biallelic Hamlin sweet orange mutant lines in the EBE were generated. The biallelic mutants are resistant to *Xcc*. Biallelic mutation of the EBE region abolishes the induction of *CsLOB1* by *Xcc*. This study represents a significant improvement in sweet orange gene editing efficacy and generating disease-resistant varieties *via* CRISPR-mediated genome editing. This improvement in citrus genome editing makes genetic studies and manipulations of sweet orange more feasible.

## Introduction

Citrus is one of the most important fruit crops worldwide because of its delightful flavor and scent, as well as its health properties. Sweet orange (*Citrus sinensis*) is the most economically important species for the citrus industry. However, citrus production is facing many biotic (e.g., diseases and insects) and abiotic challenges (e.g., drought, flooding, acidity, salinity, heat, cold, drought, and nutrient deficits) (Li, 2009). Among them, citrus diseases such as citrus canker caused by *Xanthomonas citri* subsp. *citri* (*Xcc*) (Ference et al., 2018) and Huanglongbing (HLB, also known as citrus greening) caused by *Candidatus* Liberibacter asiaticus (Bové, 2006; Wang, 2019; Yuan et al., 2020) are causing devastating effects on the citrus industry. Most elite citrus varieties, including sweet orange and grapefruit varieties, are susceptible to canker and HLB disease. Genetic improvements of citrus *via* conventional approaches are challenging. Most citrus varieties result from human selection of natural mutations or natural hybridization rather than artificial hybridization. Hybridization-based breeding for citrus is hindered by long juvenile stage (3–10 years), a highly heterozygous nature, heterozygous nature of nucellar seedlings (clones of the female parent), and male or female sterility (Omura and Shimada, 2016). It has been suggested that CRISPR-mediated genome editing will revolutionize the genetic improvements of citrus and other tree crops (Dutt et al., 2020; Wheatley and Yang, 2020).

Citrus genome editing mediated by the CRISPR technology was first reported in 2014 (Jia and Wang, 2014a,b). The first biallelic mutant of citrus (Carrizo citrange, a rootstock variety) was reported in 2017 (Zhang et al., 2017a). The first biallelic/homozygous mutations of citrus scion varieties [Pummelo (*Citrus maxima*)] were reported in 2020 (Jia and Wang, 2020). It is noteworthy that Pummelo is highly homozygous (Wu et al., 2018) and relatively easy to generate biallelic/homozygous mutants compared to other citrus species. However, most elite citrus varieties including sweet orange are highly heterozygous and biallelic/homozygous mutants have not been reported to date or showed very low editing efficiency (Jia et al., 2017a,2019a; Peng et al., 2017). We aimed to further improve the CRISPR/Cas system to make genome editing of elite citrus varieties more achievable and improve the disease resistance of sweet orange against canker. Utilization of resistant varieties is the most efficient and eco-friendly approach to control diseases. However, most commercial citrus varieties including sweet orange are susceptible to citrus canker (Favaro et al., 2020; Ference et al., 2020).

Improvement of genome editing efficiency of plants has been conducted *via* multiple approaches including improvements of the expression of Cas proteins and sgRNA using different promoters. Several promoters for driving Cas9 expression have been tested, such as 35S promoter, ubiquitin promoter (Castel et al., 2019), cell division-specific Yao promoter (Yan et al., 2015), egg cell-specific EC1.2/DD45 (Wang et al., 2015), and RPS5a promoter expressing at early embryonic stage (Tsutsui and Higashiyama, 2017; Ordon et al., 2020). Among them, the ubiquitin promoter and RPS5a promoter have significantly improved genome editing efficacy compared to the commonly used 35S promoter in Arabidopsis, tomato, rice, or maize (Stavolone et al., 2003; Cermak et al., 2017; Tsutsui and Higashiyama, 2017; Castel et al., 2019). Endogenous polymerase III promoters (U3 or U6) improve the expression of sgRNAs (Sun et al., 2015; Qi et al., 2018). In addition, other approaches including codon optimization of the Cas proteins (Li et al., 2014) and temperature optimization (Xiang et al., 2017) have shown promises to improve the efficacy of genome editing.

*Xanthomonas citri* subsp. *citri* causes the characteristic hypertrophy and hyperplasia symptoms on citrus tissues *via* secretion of PthA4, a transcriptional activator-like (TAL) effector, through the type III secretion system (Swarup et al., 1991; Yan and Wang, 2012; An et al., 2020). PthA4 enters the nucleus and activates the expression of the canker susceptibility gene *LATERAL ORGAN BOUNDARIES 1* (*LOB1*) *via* binding to the effector binding element (EBE) in the promoter region using its central nearly identical tandem repeats of 33–34 amino acids (Hu et al., 2014). The specific recognition between nucleotides and the repeat is determined by the 12th and 13th amino acids of each repeat, known as the “repeat-variable diresidue” (RVD) (Moscou and Bogdanove, 2009; Römer et al., 2009). Mutation of the EBE regions has been used to generate disease resistance crops against TAL effector-dependent pathogens such as bacterial blight of rice (Li et al., 2012; Oliva et al., 2019). In our previous study, genome modified Duncan grapefruit of the coding region of *LOB1* demonstrates canker resistance (Jia et al., 2017b). Mutation of both alleles of the EBE region of *LOB1* is required to abolish its induction by PthA4 and generate canker-resistant citrus plants (Jia et al., 2016, 2021; Jia and Wang, 2020). However, biallelic/homozygous mutation of the EBE region for elite varieties such as sweet orange is yet to be achieved. Most importantly, highly efficient generation of biallelic/homozygous mutation in elite varieties such as sweet orange and grapefruit has not been reported yet.

In this study, we improved the CRISPR/Cas9 system, which was used to generate six biallelic *CsLOB1* EBE mutant lines of Hamlin sweet orange. All biallelic *CsLOB1* EBE mutant plants (100%) are resistant against *Xcc*, representing a milestone in canker resistance development for elite citrus varieties. In addition, this improved CRISPR/Cas9 system efficiently (100%) edits the tobacco genome. Hence, this improved CRISPR/Cas9 system may be applied to other dicots besides citrus and tobacco.

## Results

### Improvement of CRISPR/Cas9 System for Citrus Genome Editing

First, we sought to improve the CRISPR/Cas9-mediated genome editing efficiency in citrus using the phytoene desaturase (*PDS*) gene as a target. The *PDS* gene was chosen for editing because of the obvious albino phenotype caused by homozygous or biallelic knockout mutation of the *PDS* gene (Zhang et al., 2017a; Huang et al., 2020). The albino phenotype serves as the functional editing (null mutation) readout. We first edited Carrizo citrange, a hybrid between *C. sinensis* “Washington” sweet orange and *Poncirus trifoliata*, which can be relatively easily transformed (compared to sweet orange) and edited based on our previous experience (Jia et al., 2017a; Huang et al., 2020). A green fluorescence (GFP) visual maker was included in the constructs to further confirm successful plant transformation in addition to antibiotic marker selection. Inspired by previous reports showing that increased culture temperature can increase editing efficacy (LeBlanc et al., 2018; Malzahn et al., 2019; Milner et al., 2020), we sought to test if we can increase editing efficacy by increasing culture temperature. The editing efficiency was higher at 30°C for both gene editing in protoplasts (Supplementary Figure 1A and Supplementary Table 4) and in *Agrobacterium*-mediated transformed epicotyl tissue than that at room temperature (Supplementary Figure 1).

**FIGURE 1:**
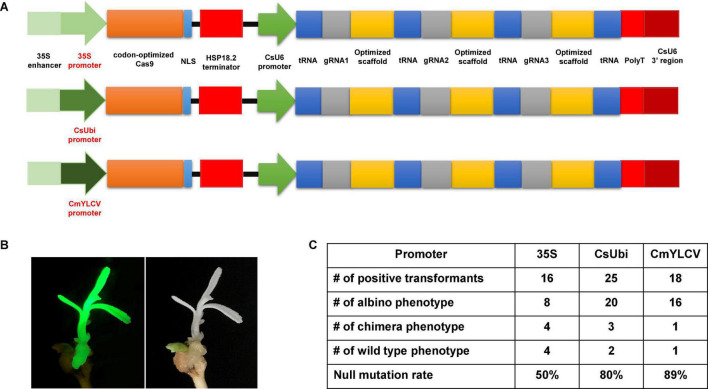
Optimal promoters for driving Cas9 expression to improve gene editing efficacy in citrus. **(A)** 35S promoter, citrus ubiquitin promoter (CsUbi), and *Cestrum yellow leaf curling virus* (CmYLCV) promoter for driving Cas9 expression in the CRISPR constructs. **(B)** A representative picture showing albino phenotype, caused by null mutation of the *PDS* gene with CmYLCV promoter construct. The construct contains GFP as visual marker. **(C)** Summary of editing efficacy from three constructs shown in **(A)**.

Previously, we improved the CRISPR/Cas9 system by codon optimizing the Cas9 gene, identifying citrus U6 (CsU6) promoter, using an improved gRNA scaffold, and inclusion of tRNA for multiplexing (Xie et al., 2015; Huang et al., 2020). Here we aimed to select a superior promoter for driving Cas9 expression. The 320 bp core region of the 35S promoter in the CRISPR/Cas9 construct was replaced with either the *Citrus sinensis* ubiquitin (CsUbi) or *Cestrum yellow leaf curling virus* (CmYLCV) promoter (Supplementary Data 1). The CsUbi promoter (Cs4g11190) was selected through mining of citrus RNAseq data (Ribeiro et al., 2021). Cs4g11190 is highly expressed in various stages of leaf development (Supplementary Table 1). The promoter region of CsUbi (Cs4g11190) was amplified from the citrus genome. The CmYLCV promoter (Stavolone et al., 2003) is a strong promoter and functions in both monocots and dicots. The upstream 538 bp regulatory region containing the enhancer elements of 35S was kept and fused with candidate promoters (Figure 1A) to enhance Cas9 expression and/or promote early expression of Cas9 in first dividing cells (Ow et al., 1987; Benfey et al., 1990). The editing efficacy of 35S, CsUbi, and CmYLCV driving Cas9 expression at 30°C was compared (Figure 1A). The albino phenotype was used as a readout for null mutations (Figure 1B). The 35S promoter produced 50% albino plants; the CsUbi promoter and CmYLCV promoter generated 80 and 89% albino plants, respectively (Figure 1C and Supplementary Figure 2), suggesting that CsUbi and CmYLCV promoters are superior to 35S for gene editing in citrus.

In all transformation events, we observed chimeric albino phenotype. It is common to observe chimera phenotypes during plant transformation of explants, such as epicotyledons, leaves, and stems. The chimera rate for the 35S, CsUbi, and CmYLCV was 25, 12, and 5.6%, respectively (Figure 1C). These results suggest that CsUbi and CmYLCV promoters drive an early expression of the Cas9 in the first transformed cells compared to 35S promoter. All constructs contained GFP. Not surprisingly, GFP florescence was observed to colocalize with albino phenotype. Therefore, for all the transformation experiments, only transformants with evenly distributed GFP florescence all over the plantlets were kept for further analyses.

### The Improved CRISPR/Cas9 System Efficiently Edits Tobacco (*Nicotiana tabacum*)

**FIGURE 2:**
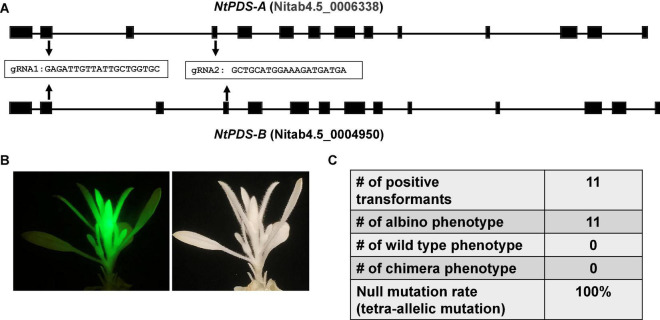
The improved CRISPR/Cas9 system works efficiently for genome editing of tobacco (*Nicotiana tabacum*). **(A)** Gene structures of two *NtPDS* homologs from allotetraploid *Nicotiana tabacum*. Two gRNAs conserved in both *NtPDS-A* and *NtPDS-B* genes as indicated were designed for genome editing. **(B)** A representative picture shows the albino phenotype in genome modified *Nicotiana tabacum*. The construct contains GFP as visual marker. **(C)** Summary of editing efficacy.

The CmYLCV promoter is a strong constitutive promoter for heterologous gene expression in *Arabidopsis thaliana* and *Nicotiana tabacum* and in a wide variety of crops including *Lycopersicon esculentum*, *Zea mays*, and *Oryza sativa* (Stavolone et al., 2003). We hypothesized that the improved CRISPR/Cas9 system driven by CmYLCV works in other plant species, especially dicots. To test this hypothesis, we tested the construct in tobacco (*N. tabacum*), an allotetraploid species, considering its amenability to stable transformation. There are two *PDS* homologs, *PDS-A* and *PDS-B* in tobacco genome. Two gRNAs were designed in the conserved regions between the two homologs (Figure 2A). We obtained a total of 11 transformants with GFP. The transformants with green fluorescence all showed albino phenotype (Figure 2B and Supplementary Figure 3), indicating that we achieved tetra-allelic mutations for *PDS-A* and *PDS-B* in all positive transformants (Figure 2C). This result demonstrates that the improved CRISPR/Cas9 system achieves a 100% null mutation efficacy for *PDS* genes in tobacco. This result suggests that our improved CRISPR/Cas9 system may be able to efficiently edit genomes of other dicots, besides citrus and tobacco.

### Highly Efficient Generation of Biallelic Mutation in Sweet Orange

Sweet orange is a hybrid between pummelo (*C. maxima*) and mandarin (*Citrus reticulata*), and most genes have two different alleles. Here, we used the improved CRISPR/Cas9 system that contains a codon optimized Cas9, tRNA for multiplexing (Xie et al., 2015; Huang et al., 2020), CsU6 promoter (Huang et al., 2020), improved sgRNA scaffold (Dang et al., 2015), and the CmYLCV promoter to edit the EBE in the promoter region of the canker susceptibility gene *CsLOB1* (Hu et al., 2014). The EBE region of sweet orange contains two different types/alleles. We aimed to edit both alleles to create null mutations.

**FIGURE 3:**
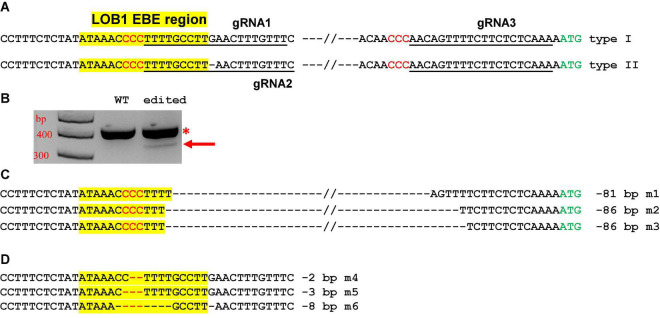
*CsLOB1* editing in Hamlin sweet orange protoplasts. **(A)** *CsLOB1* promoter contains two different types. The EBE region was highlighted with yellow; regions chosen for gRNAs were underlined; PAM was shown in red. The final CRISPR constructs contains three gRNAs as indicated. **(B)** PCR products generated by two primers spanning the EBE and last gRNA. The PCR product with an arrow was amplified from the deletion mutants. **(C)** Sanger sequencing results of the PCR product with the arrow in **(B)**. Twenty clones were sequenced. m1, 11 clones; m2, 5 clones; m3, 4 clones. **(D)** Sanger sequencing results of the PCR products marked by * in **(B)** that were cloned for colony sequencing. Ninety-four clones were sequenced. m4, 2 clones; m5, 2 clones; m6, 1 clone; WT, 89 clones.

Two different gRNAs for both type I and type II EBEs were designed. A third gRNA targeting a region upstream of the translation start codon ATG was also included (Figure 3A). The final construct contains these three gRNAs. Such a design aimed to create not only small indels, but also longer deletion mutations. We first transformed citrus protoplasts to confirm the functionality of the construct. As expected, the construct not only produced indel mutations, but also generated relatively long deletion mutations (Figures 3B–D).

**FIGURE 4:**
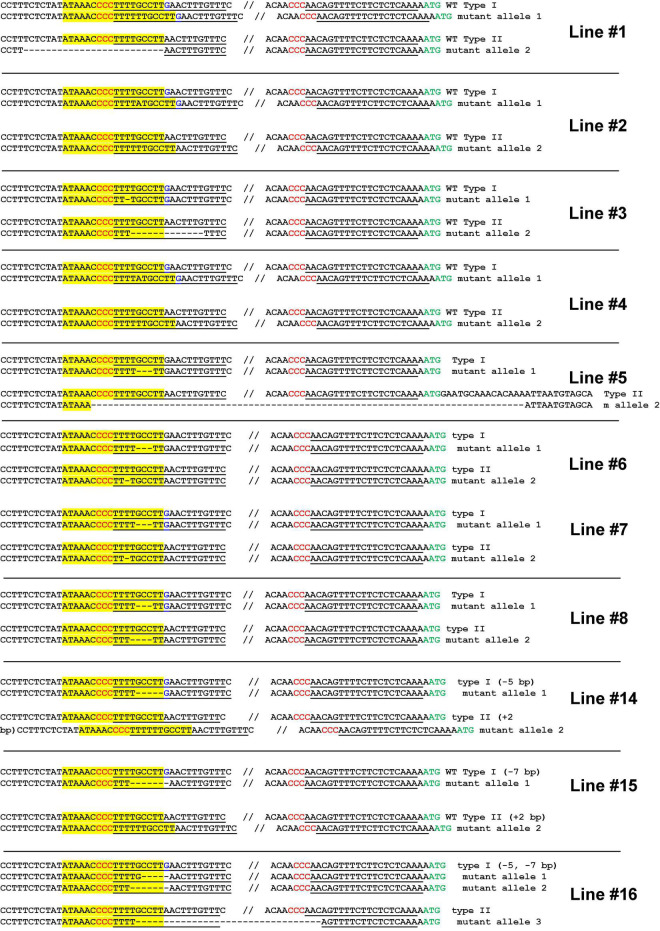
Highly efficient generation of biallelic mutant citrus plants for Hamlin sweet orange. Genotyping of biallelic mutants. Mutations of the EBE region is the focus of the genotyping. The EBE region was highlighted with yellow; regions chosen for gRNAs were underlined; PAM was indicated in red; nucleotide G in blue is a SNP only in one allele, but not in another allele.

Next, we performed a stable transformation of Hamlin sweet orange epicotyls. A total of 16 positive transformants were obtained. Two lines died during regeneration. Finally, we obtained 14 transgenic plants. We genotyped these 14 seedlings. Among them, 11 plants contained biallelic mutations in the EBE region (Figure 4 and Supplementary Figure 4), representing a 78.6% biallelic mutation rate (Supplementary Table 2). The three heterozygous mutants contained both wild-type and edited EBEs. Subsequently, we micro-grafted these seedlings onto the rootstock variety Carrizo citrange. A total of six biallelic mutants and two heterozygous mutants were successfully grafted on the rootstock, whereas the rest did not survive.

### Biallelic Mutants Are Resistant to *Xanthomonas citri* subsp. *citri*

**FIGURE 5:**
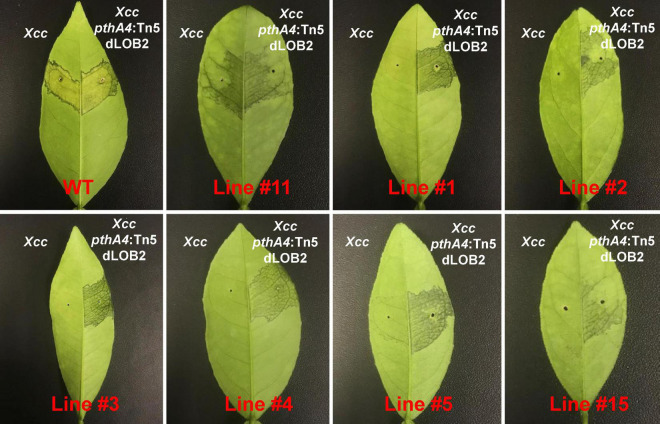
Biallelic mutants of the EBE region of *CsLOB1* of Hamlin sweet orange are resistant to *Xcc. Xcc* inoculation assay was conducted using fully expanded young leaves. Line #11 is a heterozygous mutant; other lines are biallelic mutants. For each line, left half of the tested leaf was syringe inoculated with wild-type *Xcc* (10^8^ CFU/ml); the right half of the tested leaf was inoculated with *Xcc pthA4*:Tn5 carrying designer TAL effector dLOB2 (10^8^ CFU/ml). Pictures were taken at 8 days post inoculation.

We hypothesized that the biallelic mutation of the EBE region of *CsLOB1* disrupts its binding to *Xcc* TAL effector PthA4, thus *Xcc* is unable to induce canker susceptibility gene *CsLOB1* for symptom development (Swarup et al., 1992; Hu et al., 2014). To test this hypothesis, wild-type Hamlin sweet orange plants, one heterozygous mutant line (line #11) and six biallelic mutant lines (line #1, 2, 3, 4, 5, 15) of Hamlin sweet orange were tested for resistance against *Xcc* strains (Figure 5). For each genotype, one half of the leaf was inoculated with wild-type *Xcc*, and another half was inoculated with *Xcc pthA4*:Tn5 mutant carrying the designer TALE dLOB2, which activates the expression of *LOB2* (Teper et al., 2020). *LOB2* is a *LOB1* homolog that causes canker symptoms when artificially induced, such as in the presence of dLOB2 (Zhang et al., 2017b; Teper et al., 2020). *Xcc pthA4*:Tn5 dLOB2 was included as a control. The onset of canker symptoms by *Xcc pthA4*:Tn5 dLOB2 on the same leave can exclude the possibility that lack of canker symptoms for biallelic mutants is owing to leaf age. At 8 dpi, all wild-type Hamlin leaves developed canker lesions for both wild-type *Xcc* and dLOB2 carrying *Xcc pthA4*:Tn5. No canker symptoms were observed for the biallelic mutant lines (line #1, 2, 3, 4, 5, and 15) when inoculated with wild-type *Xcc*. On the contrary, canker symptoms were observed on the other half of the leaves inoculated with *Xcc pthA4*:Tn5 dLOB2. The heterozygous mutant line #11 showed canker disease symptoms for both wild-type *Xcc* and dLOB2 carrying *Xcc pthA4*:Tn5 (Figure 5). Taken together, the present results demonstrate that the biallelic Hamlin sweet orange mutant lines are resistant to *Xcc*.

### *Xanthomonas citri* subsp. *citri* Does Not Induce the Expression of *CsLOB1* in Biallelic Mutants

**FIGURE 6:**
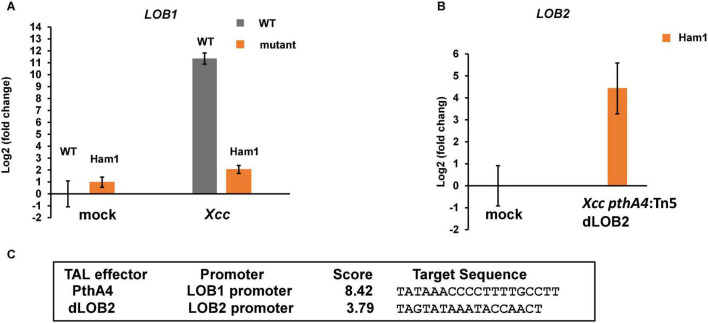
Induction of *CsLOB1* by *Xcc* is abolished in a biallelic mutant line of Hamlin sweet orange. **(A)** RT-qPCR analyses of *CsLOB1* relative expression in leaf samples collected at 48 h post inoculation with indicated treatments. Mock, mock inoculation with buffer; *Xcc*, inoculation with wild-type *Xcc* (10^8^ CFU/ml); dLOB2, inoculation with *Xcc pthA4*:Tn5 carrying designer TAL effector dLOB2 (10^8^ CFU/ml). Each treatment has three biological repeats. Ham1, a representative biallelic mutant line #1 (aka Ham1). All expression levels were normalized to the WT mock. The GAPDH gene was used as an endogenous control. **(B)** RT-qPCR analysis of *LOB2* relative expression of leaf samples collected at 48 h post inoculation with indicated treatment. All expression levels were normalized to the Ham1 mock. The GAPDH gene was used as an endogenous control. **(C)** Finding promoter binding site for TAL effectors by using Target Finder (https://tale-nt.cac.cornell.edu/node/add/talef-off). Designer TAL effector dLOB2 shows affinity to the *LOB2* promoter, but not the LOB1 promoter.

Next, we tested whether biallelic mutations of the EBE region of *CsLOB1* abolish its induction by *Xcc* using reverse transcription-quantitative PCR (RT-qPCR). The expression level of *CsLOB1* in wild-type Hamlin sweet orange was dramatically induced when inoculated with *Xcc*, which is consistent with previous findings (Hu et al., 2014; Teper et al., 2020). The induction of *CsLOB1* was obliterated in the biallelic mutant line Ham1 (Figure 6A). *Xcc pthA4*:Tn5 dLOB2 induced the expression of *LOB2* (Figures 6B,C), which is consistent with canker symptoms induced by *Xcc pthA4*:Tn5 dLOB2 on both wild-type and biallelic mutant lines (Figure 5) and with our previous result (Teper et al., 2020).

### Analysis of Off-Targets

To investigate whether this improved CRISPR/Cas9 can introduce off-target mutations, we amplified and sequenced 20 potential off-target sites with 4 or fewer mismatches within the protospacers in all 6 canker-resistant Hamlin mutant lines. No potential off-targets containing less than 4 mismatches were found. Hence, it is very likely that there are no off-targets. The sequencing results showed that no mutations at potential off-target sites were detected (Supplementary Table 5).

## Discussion

In this study, we improved CRISPR/Cas9 system to achieve high efficacy for biallelic editing in citrus. The improved system contains a dicot codon-optimized Cas9, tRNA for multiplexing, an improved sgRNA scaffold with high efficiency (Dang et al., 2015), CsU6 to drive sgRNA expression, and superior promoters (CmYLCV or CsUbi promoter). The dicot codon-optimized Cas9 can work efficiently in tomato (Cermak et al., 2017), citrus, and *N. benthamiana*. The improved sgRNA scaffold contains a shorter T-stretch to avoid early termination and 5 nt extensions of the tetraloop for stability (Dang et al., 2015). Importantly, this improved CRISPR/Cas9 system has significantly increased the biallelic/homozygous mutation rates. For Carrizo citrange, the CsUbi and CmYLCV driven constructs achieved null mutation rates of 80 and 89%, respectively, *via* epicotyl transformation. In addition, the CmYLCV construct attained a 79% biallelic mutation rate in Hamlin sweet orange even under restrained gRNA options (EBE region). The difference in editing efficacy may also result from the differential transformation efficacy in different citrus genotypes. The transformation efficacy was reported to be 20.6% for Carrizo citrange (Pena et al., 1995), but ranged from 0.88 to 23.8 for sweet orange varieties (Poles et al., 2020) and was 2.5% for Hamlin sweet orange (Fávero et al., 2012). Previously, the biallelic mutation rate was reported to be 5.3% for grapefruit (Jia et al., 2021), 44.4% for Carrizo citrange (11.1% homozygous mutation rate) (Huang et al., 2020), and 22.7–55% for Carrizo citrange (Zhang et al., 2017a). Thus, this improved CRISPR/Cas9 system we reported here represents a significant improvement compared to previous studies. In addition, this new system uses a citrus U6 promoter and a CmYLCV promoter to drive sgRNA or Cas9 protein, respectively. It has been recommended to use endogenous promoters to drive Cas proteins or sgRNA to improve efficacy or to address gene regulation concerns. It was reported that U6 promoters and two ubiquitin promoters identified in grapevine significantly increase the editing efficiency in grape by improving the expression of sgRNA and Cas9, respectively (Ren et al., 2021). Similarly, endogenous promoters for U6 and ubiquitin were successfully used for genome editing of maize and yielded mutation efficiencies ranging from 48.5 to 97.1% (Qi et al., 2018). The improved CRISPR/Cas9 system developed in this study can be used not only for editing the genome of citrus, but also for other plant species. We successfully edited tobacco *PDS* genes (100% null mutation) with the CmYLCV-driven construct. We anticipate that the modified CRISPR/Cas9 system we developed is efficient in genome editing of other plant species, especially dicots.

The CmYLCV promoter and CsUbi promoter driving the expression of Cas9 have significantly improved the citrus editing efficacy compared to that of the 35S promoter. It has been reported that strong and constitutive promoters isolated from the plant housekeeping genes such as ubiquitin or actin can achieve higher mutation rates in various monocot and dicot species (Hassan et al., 2021). The CmYLCV promoter was reported to function in both dicots and monocots and shows higher or comparable activities than the 35S promoter in driving the heterozygous expression (Stavolone et al., 2003). The CmYLCV promoter may drive early expression of the Cas9 in the first transformed cells as suggested by the fewest chimera observed. Similar to the 35S promoter, the CmYLCV promoter is an RNA polymerase II promoter. The CmYLCV promoter demonstrates similar efficacy as the Ubiquitin 2 promoter of switchgrass in transformation, reaching about 80 and 100% of protoplasts after 24 and 48-h, respectively, post transformation (Weiss et al., 2020). In addition to driving the expression of Cas proteins, the CmYLCV promoter has also been successfully used to drive the sgRNA array *via* Csy4-mediated processing or gRNA–tRNA array (Cermak et al., 2017).

We successfully obtained multiple biallelic canker-resistant Hamlin sweet orange mutant lines against *Xcc*, which is a TAL effector-dependent pathogen, *via* editing the EBE region of canker susceptibility gene *LOB1* (Hu et al., 2014). TAL effectors are critical virulence factors for most *Xanthomonas* pathogens (Boch and Bonas, 2010; Perez-Quintero and Szurek, 2019; An et al., 2020). TAL effectors are known to be subject to repeated recombination that allows them to recognize new targets or overcome the resistance resulting from mismatches between the TAL effectors and the EBE regions. It was reported that *Xcc* is able to overcome 2–5 mismatches between the repeat region of PthA4 and the EBE of *CsLOB1*, but is unable to overcome the resistance when the mismatch is equal or more than 7 (Teper and Wang, 2021). However, the previous study was conducted *via* an accelerated evolutionary approach that includes injection with high titers of *Xcc* than normally encountered in natural settings. In addition, the TALEs were cloned into pBBR1MCS5, a medium copy number vector (approximately 30 copies), whereas naturally occurring PthA4 is three copies in each bacterial cell (Teper and Wang, 2021). It is anticipated that the chance for *Xcc* to overcome the mismatch between PthA4 and the EBE region in natural settings is much lower than that observed *via* the accelerated evolutionary study (Teper and Wang, 2021). Additionally, the genome modified lines will enable us to investigate whether *Xcc* can overcome the resistance resulting from EBE mutations in natural settings in future studies. The biallelic mutants also can serve as a diagnostic tool to monitor emerging *Xcc* strains that target other susceptibility genes such as *LOB2* and *LOB3* (Zhang et al., 2017b; Teper et al., 2020). A similar strategy has been developed for *Xoo*/rice pathosystem (Eom et al., 2019).

Of note, the canker-resistant sweet orange lines contain the CRISPR/Cas construct, which is considered as transgenic and might not be suitable to be used for citrus production. Nevertheless, successful generation of canker-resistant sweet orange represents a milestone in the ultimate utilization of canker-resistant citrus varieties based on mutations of the coding or the EBE region of the canker susceptibility gene *LOB1*. Generation of canker-resistant citrus varieties will overcome the significant issues in canker management using copper-based products (Zhang et al., 2003), antibiotics (Hu and Wang, 2016; Li et al., 2019, 2020), and biocontrol bacteria. Such an approach has been successfully used to generate disease-resistant rice varieties (Oliva et al., 2019). For rice, the CRISPR/Cas construct can be easily removed by progeny segregation or back-crossing, which is not practical for citrus. This is because citrus has long juvenile stage. In addition, back-crossing leads to the loss of the elite quality of parental varieties. In future studies, we will aim to generate non-transgenic genome-modified canker-resistant citrus varieties *via* transient expression of the CRISPR/Cas construct. Such a method has been successfully used to generate non-transgenic genome-edited crops (Zhang et al., 2016; Chen et al., 2018; Lin et al., 2018; Veillet et al., 2019). Development of methods using *in vitro* assembled CRISPR/Cas9/gRNA ribonucleoprotein (Cas9/gRNA RNP) complexes for transient expression of the CRISPR/Cas9 system in citrus may also be an attractive future option given that this approach has proven successful in producing other crop plants that contain precisely edited genes and that are non-transgenic (Woo et al., 2015; Svitashev et al., 2016; Zhang et al., 2021). In addition, newly emerging DNA delivery technologies such as nanoparticles (Demirer et al., 2019; Lv et al., 2020) might help deliver our highly efficient constructs to achieve non-transgenic genome editing.

## Materials and Methods

### Plant Materials

The seeds of Carrizo citrange were purchased from Lyn Citrus Seeds (Arvin, CA, United States). The seeds of sweet orange Hamlin were harvested from mature fruits in the groves of the Citrus Research and Education Center, University of Florida.

### Constructs

To test the performance of different promoters for driving Cas9, we replaced the 320 bp core region of the 35S promoter in construct pC-CsU6-tRNA-3PDS, which was reported previously (Huang et al., 2020), with promoters used in this study. Specifically, the 35S promoter sequence was digested with *Bsp*EI and *Sal*I from pC-CsU6-tRNA-3PDS. CsUbi or CmYLCV (Supplementary Data 1) was PCR amplified and inserted into the same site to replace the 35S promoter. PC-CsUbi-PDS and PC-CmYLCV-PDS constructs were obtained and confirmed by Sanger sequencing. To make the *CsLOB1* EBE CRISPR construct, primers LOBPro3-F1/R1 and LOBPro3-F2/R2 (Supplementary Table 3) were used to make multiplex constructs using the transient vector pXH1-CsU6-tRNA *via* Golden gate cloning as described previously (Huang et al., 2020). Three gRNAs were included: one targeting type I EBE region of *CsLOB1*, one targeting type II EBE region of *CsLOB1* (Jia et al., 2016, 2017b; Jia and Wang, 2020), and one targeting a region upstream of ATG. The construct PXH1-LOB1-Pro3 was confirmed by Sanger sequencing. The CmYLCV promoter sequence (Supplementary Data 1) was cut from PC-CmYLCV-PDS with *Sph*I + *Sal*I and inserted in the same site of PXH1-LOB1-Pro3 to replace the 35S promoter to make the transient expression construct PXH1-CmYLCV-LOB1-Pro3. This construct was used for protoplast transfection.

For stable transformation, the cassette was cut from PXH1-CmYLCV-LOB1-Pro3 with *Fsp*I and *Xba*I and inserted into the *Zra*I/*Xba*I site of the binary vector PCXH2, which carries the GFP expression cassette to make the final construct PCXH2-CmYLCV-LOB1-Pro3. This construct, after transforming into *Agrobacterium* strain EHA105, was used for *Agrobacterium*-mediated citrus epicotyl transformation.

For tobacco *NtPDS* gene editing, we also used the same tRNA-based multiplex vector as described above. The Cas9 is driven by CmYLCV promoter, and the gRNAs for *NtPDS* are driven by CsU6 promoter. Two gRNAs were designed (gRNA1: GAGATTGTTATTGCTGGTGC and gRNA2: GCTGCATGGAAAGATGATGA) based on the conserved regions between the two *PDS* homologs in the genome of allotetraploid species *N. tabacum*. The final CRISPR construct was transformed into *Agrobacterium* strain EHA105, which was used for stable transformation of tobacco leaf discs.

### Protoplast Transformation

Transformation of CRISPR constructs into embryogenic citrus protoplasts was performed as described previously (Huang et al., 2020).

### Citrus Transformation

*Agrobacterium*-mediated transformation of citrus epicotyl was performed by following the protocol described previously (Jia et al., 2019b; Huang et al., 2020). After co-cultivation of epicotyls with *Agrobacterium*, the transformed epicotyls were incubated in regeneration medium at 30°C or at room temperature in the dark for 2 weeks before culturing under light at room temperature. For Carrizo *PDS* gene editing, two independent experiments were performed.

### Genotyping of Citrus Transformants

Since the transformation constructs carry the GFP expression cassette, positive transformants were selected under GFP filter in addition to antibiotic selection (Kanamycin). Citrus genomic DNA was extracted using the CTAB (cetyltrimethylammonium bromide) method (Pandey and Wang, 2019). Positive transformants were further confirmed by Cas9 amplification (primers in Supplementary Table 3). Detection of editing in the EBE region of *CsLOB1* promoter was performed by amplifying the target region (primers in Supplementary Table 3) with high fidelity DNA polymerase Q5 (New England Biolabs, Ipswich, MA, United States), followed by cloning of PCR products and sequencing.

### *Xanthomonas citri* subsp. *citri* Inoculation

*Xanthomonas citri* subsp. *citri* wild-type strain 306 and dLOB2 containing *Xcc pthA4*:Tn5 (Yan and Wang, 2012; Hu et al., 2016; Teper et al., 2020) were suspended in 20 mM MgCl2 at 10^8^ CFU/ml. The bacteria suspensions were syringe-infiltrated into young fully expanded leaves of wild-type sweet orange Hamlin sweet orange plants or mutant plants (Teper and Wang, 2021). Inoculated plants were kept in a temperature-controlled (28°C) glasshouse with high humidity. Pictures were taken 8 days post inoculation for disease resistance evaluation.

### Reverse Transcription-Quantitative PCR

Leaves of wild-type and a biallelic mutant line #1 (hereafter named Ham1) with and without *Xcc* inoculation were sampled at 48 h post inoculation. Total RNA was extracted using the TRIzol reagent (Invitrogen, Carlsbad, CA, United States) following the manufacturer’s manual. Extracted RNA was further treated with RQ1 RNase-Free DNase (Promega, Madison, WI, United States) to remove genomic DNA contamination, if any. The treated RNA was further purified with RNeasy Mini Kit (QIAGEN, Germantown, MD, United States). One microgram of RNA from each sample was used for cDNA synthesis by using QuantiTect Reverse Transcription Kit (QIAGEN). Quantitative PCR was performed using SYBR Green PCR Master Mix in QuantStudio 3 Real-Time PCR System (Applied Biosystems, Foster City, CA, United States). The citrus house-keeping gene *GAPDH* was used as an endogenous control. Quantification of gene expression was calculated by following the comparative *Ct* method (Pfaffl, 2001). Primers for RT-qPCR are listed in Supplementary Table 2.

### Analysis of Potential Off-Targets

To analyze potential off-targets, we obtained the putative off-targets using a web-based software.^1^ The potential off-target sites with four or fewer mismatches were chosen for analysis. Genomic DNA from *LOB1* EBE-edited Hamlin transgenic lines was used as template, and the primers listed in Supplementary Table 3 were used to amplify the fragments spanning the off-targets. Finally, the PCR products were subjected to Sanger sequencing to identify off-target.

## Data Availability Statement

The original contributions presented in the study are included in the article/Supplementary Material, further inquiries can be directed to the corresponding author.

## Author Contributions

XH and NW designed and organized the study. XH performed the experiments. YW contributed to off-target experiments and grafting. XH and NW prepared the manuscript.

## Conflict of Interest

The authors declare that the research was conducted in the absence of any commercial or financial relationships that could be construed as a potential conflict of interest.

## Publisher’s Note

All claims expressed in this article are solely those of the authors and do not necessarily represent those of their affiliated organizations, or those of the publisher, the editors and the reviewers. Any product that may be evaluated in this article, or claim that may be made by its manufacturer, is not guaranteed or endorsed by the publisher.

## Footnotes

1
http://crispr.hzau.edu.cn/cgi-bin/CRISPR2/CRISPR
