# Supplementary material for: Highly Efficient Generation of Canker-Resistant Sweet Orange Enabled by an Improved CRISPR/Cas9 System
Source: Front Plant Sci. 2022 Jan 11;12:769907. doi: 10.3389/fpls.2021.769907 (PMC8787272; doi:10.3389/fpls.2021.769907)
Supplement: Supplementary file 1 [file Data_Sheet_1.pdf]

Supplementary Fig. 1

A

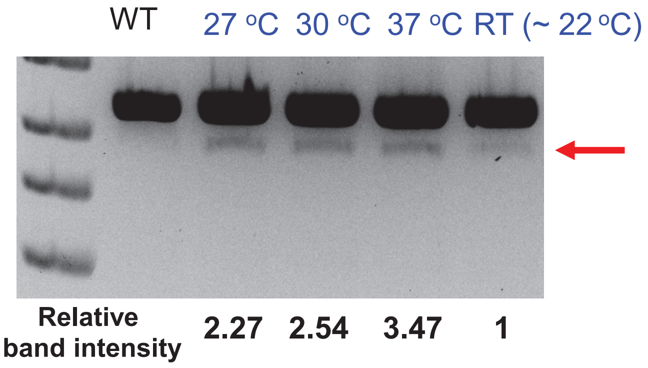

B

```
TCCGCCGCCTATAGTTCTTAGCCCTTG // TTCCACCAACCGAACCATACAAGTTCACCA LOB1 WT

TCCGCCGCCTA-----------------// -----------GAACCATACAAGTTCACCA -86 bp
TCCGCCGCCT------------------// ------------AACCATACAAGTTCACCA -88 bp
```

C

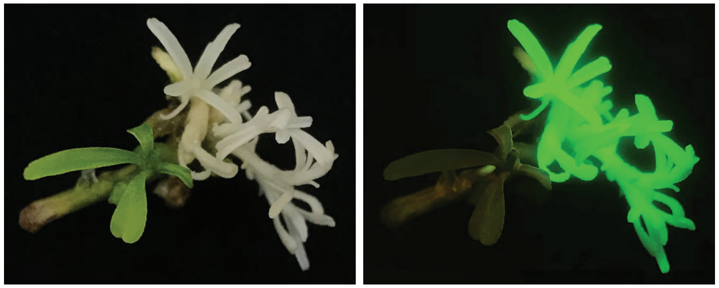

D

Temperature	RT (~22 °C)	30 °C
# of positive transformants	9	16
# of albino phenotype	4	8
# of chimera phenotype	1	4
Null mutation	44.4%	50%
Mutation rate	55.6%	75%

Supplementary Fig. 1 Higher temperatures can increase SpCas9 editing efficacy in citrus
A) CRISPR/Cas9-mediated editing of *CsLOB1* in citrus protoplasts under different temperature. Three independent assays were performed, with similar results. Numbers under the gel, relative band intensity (cleavage product with arrow) measured with ImageJ (https://imagej.nih.gov/ij/). **B)** Sanger sequencing result for PCR product indicated with the arrow in A; the sequencing data is from the sample at 30℃. **C)** A representative picture showing albino phenotype. **D)** Summary of editing efficacy at different temperature. Null mutation rate was calculated by dividing the number of pure albino plants with number of total positive transformants. Mutation rate was calculated by dividing the number of pure albino plants and chimeric plants with number of total positive transformants.

Supplementary Fig. 2

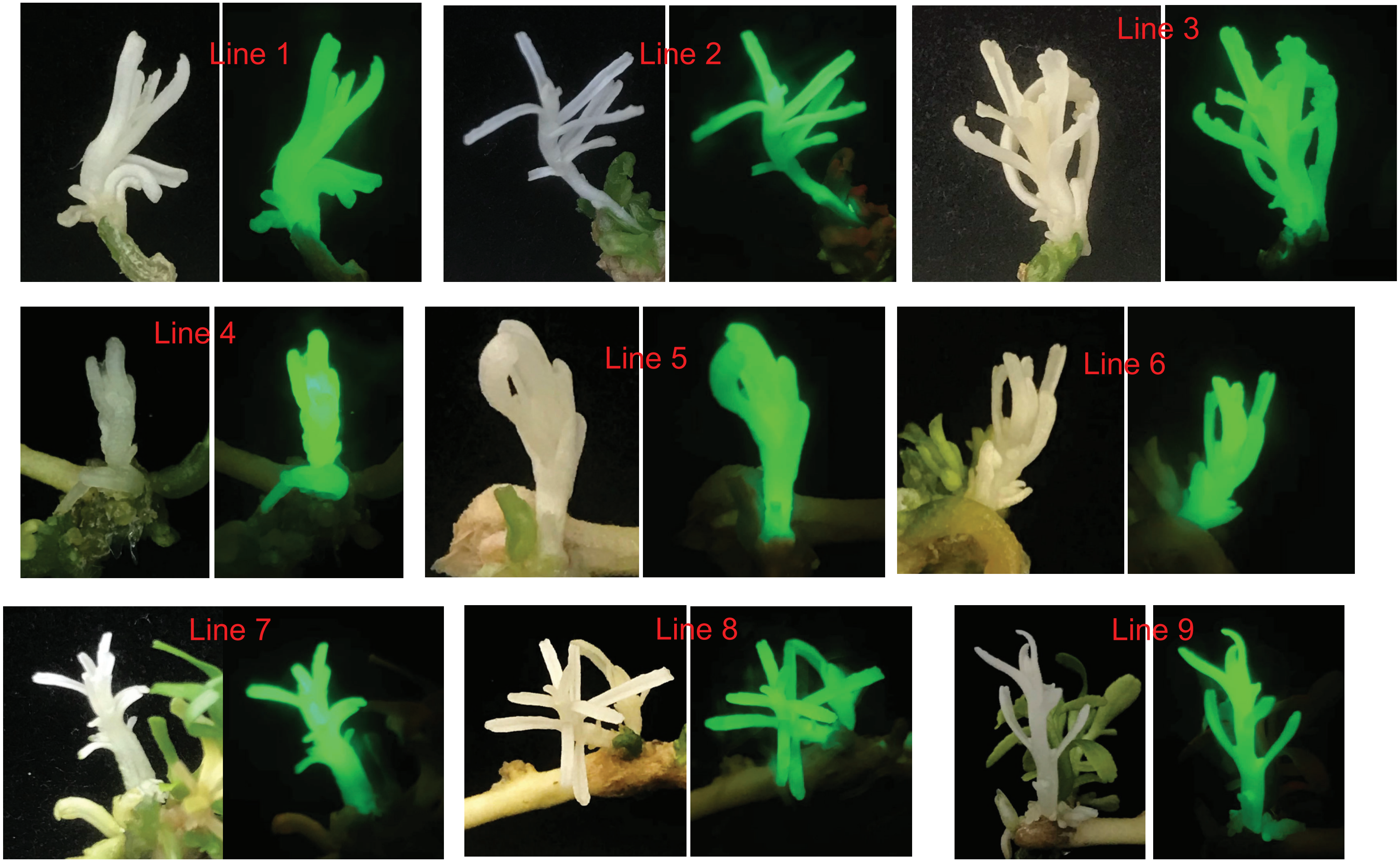

Supplementary Fig. 2

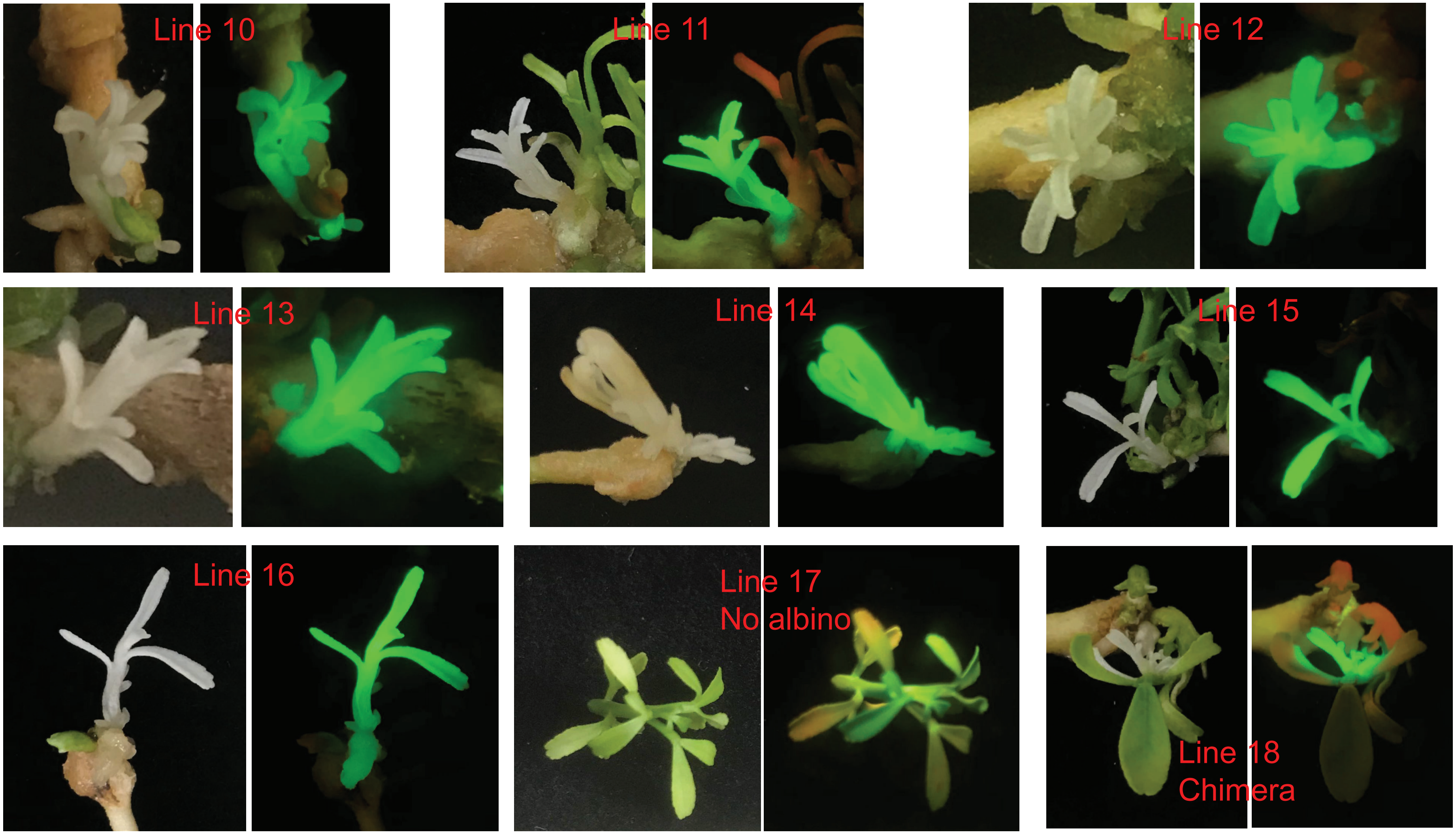

Supplementary Fig. 2 Phenotypes from CmYLCV-Cas9-PDS construct

Supplementary Fig. 3

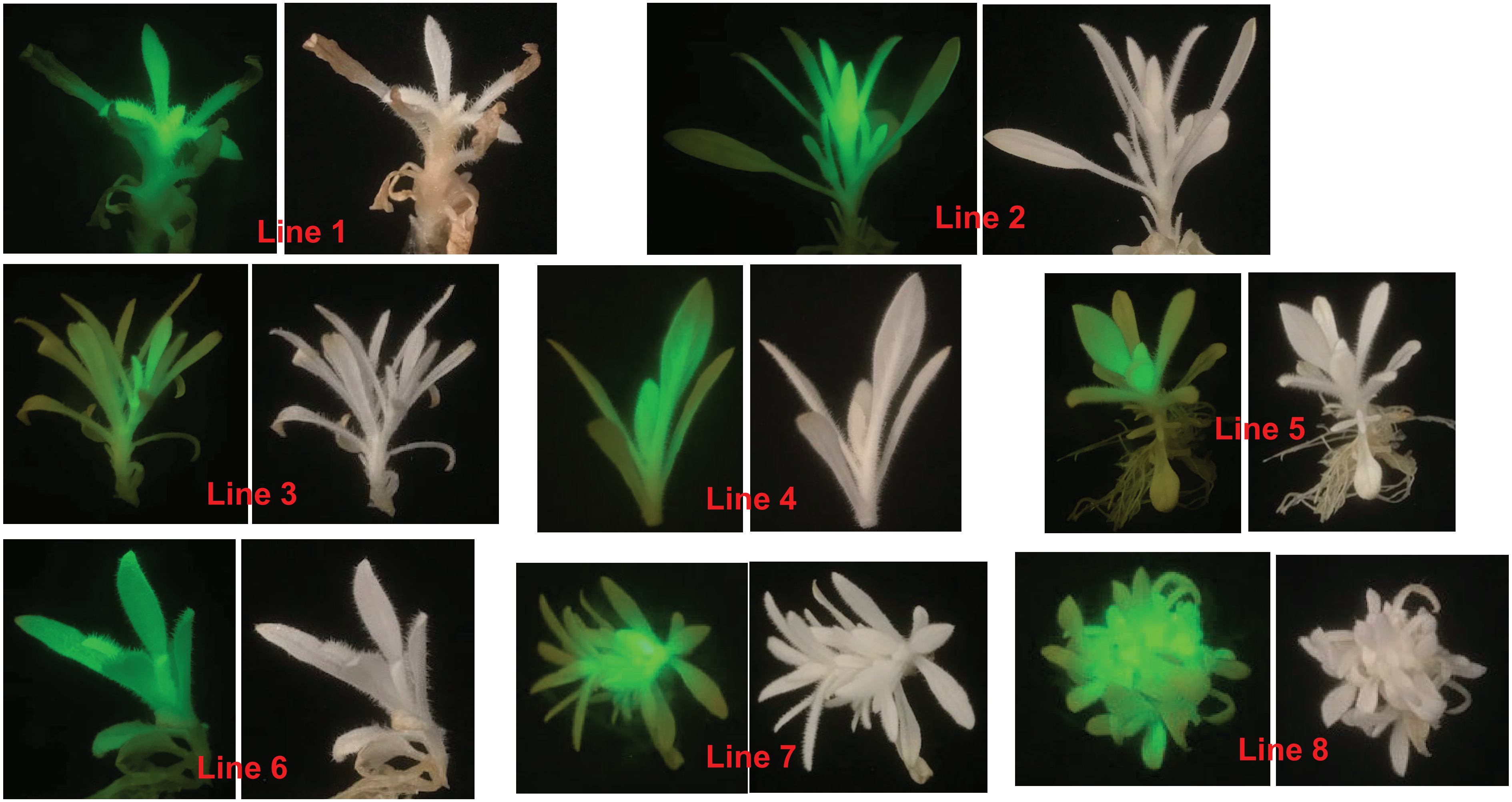

Supplementary Fig. 3

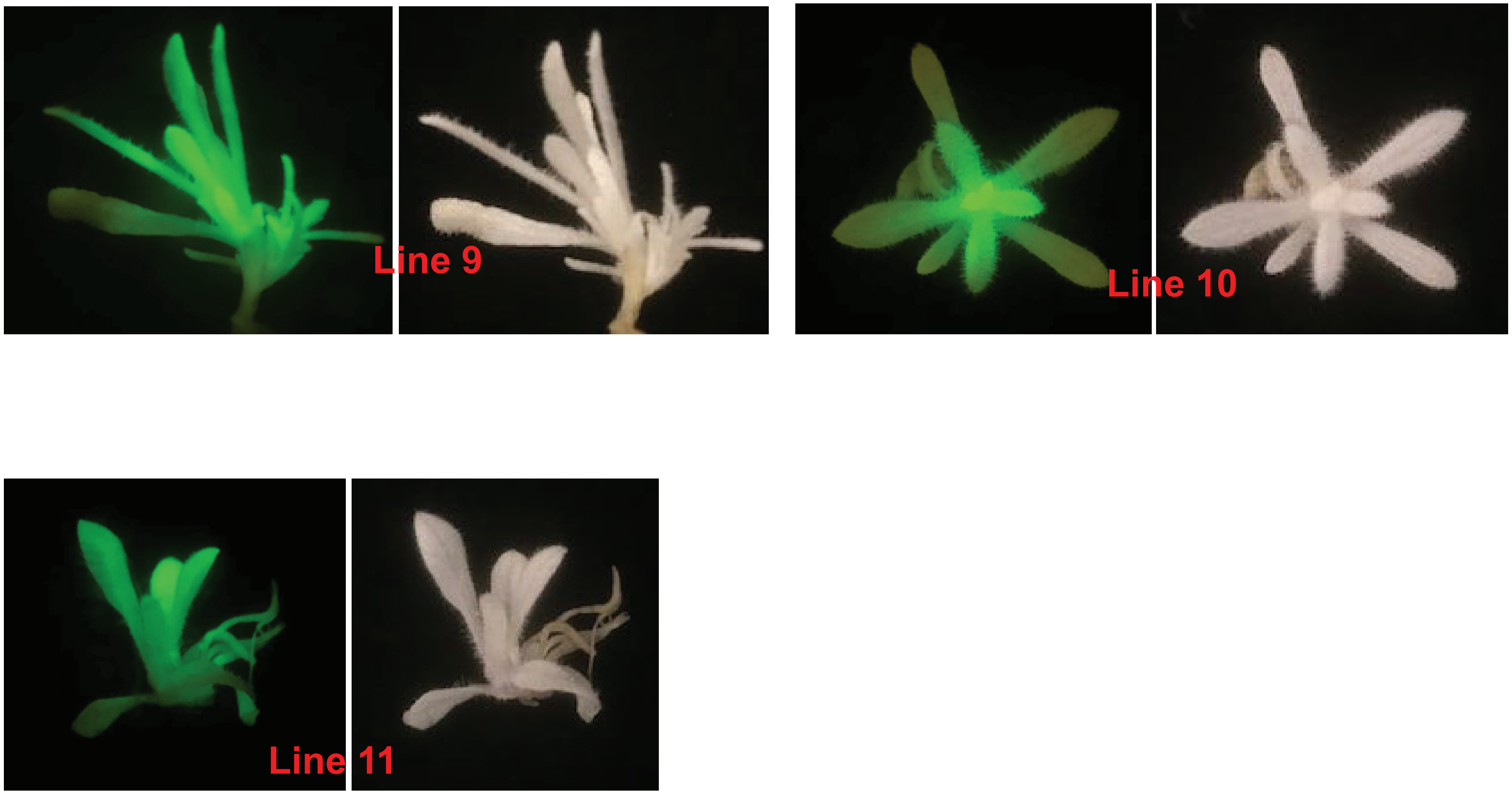

Supplementary Fig. 3 Optimized CRISPR/Cas9 system can efficiently edit *PDS* genes in tobacco (*Nicotiana tabacum*)

Supplementary Fig. 4

Line #1

```
CCTTTCTCTATATAAACCCCTTTTGCCTTGAACTTTGTTTC  //  ACAACCCAACAGTTTTCTTCTCTCAAAAATG WT Type I
                      TT
CCTTTCTCTATATAAACCCCTTTTGCCTTGAACTTTGTTTC  //  ACAACCCAACAGTTTTCTTCTCTCAAAAATG mutant allele 1

CCTTTCTCTATATAAACCCCTTTTGCCTTAACTTTGTTTC   //  ACAACCCAACAGTTTTCTTCTCTCAAAAATG WT Type II
CCTT-------------------------AACTTTGTTTC   //  ACAACCCAACAGTTTTCTTCTCTCAAAAATG mutant allele 2
```

mutant allele 1

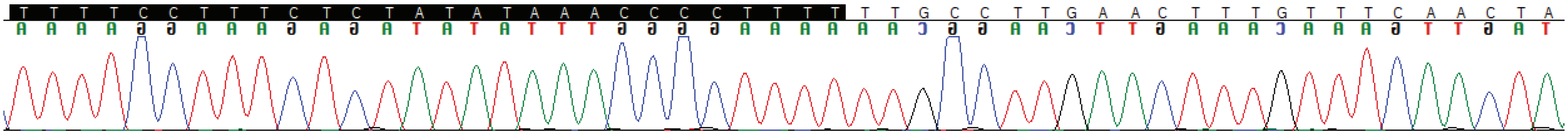

mutant allele 2

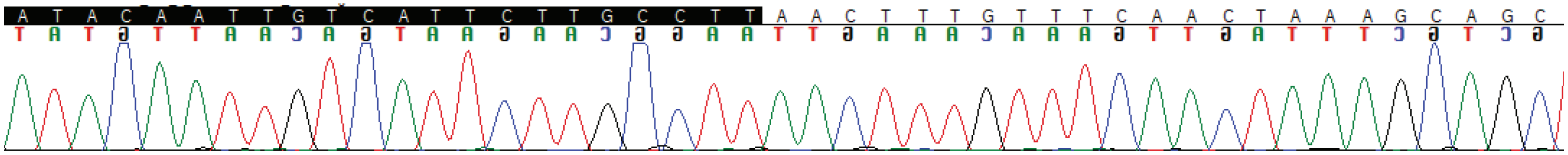

Supplementary Fig. 4

CCTTTCTCTATATAAACCCCTTTTGCCTTGAACTTTGTTTC // ACAACCCAACAGTTTTCTTCTCTCAAAAATG WT Type I

AT

CCTTTCTCTATATAAACCCCTTTTGCCTTGAACTTTGTTTC // ACAACCCAACAGTTTTCTTCTCTCAAAAATG mutant allele 1

CCTTTCTCTATATAAACCCCTTTTGCCTTAACTTTGTTTC // ACAACCCAACAGTTTTCTTCTCTCAAAAATG WT Type II

TT

CCTTTCTCTATATAAACCCCTTTTGCCTTAACTTTGTTTC // ACAACCCAACAGTTTTCTTCTCTCAAAAATG mutant allele 2

Line #2 and Line #4 same genotype

mutant allele 1

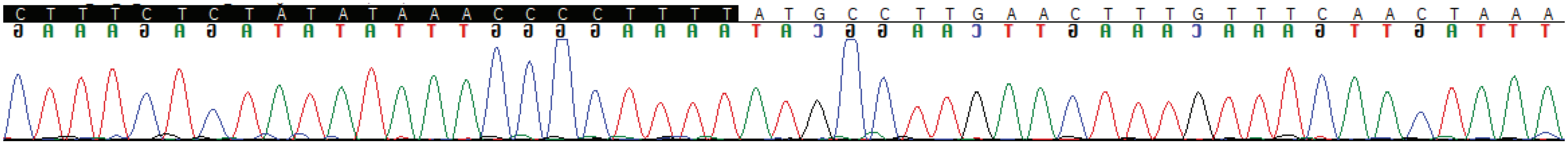

mutant allele 2

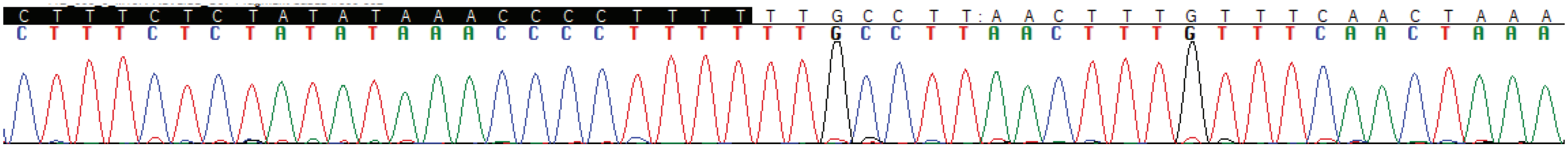

Supplementary Fig. 4

```
CCTTTCTCTATATAAACCCCTTTTGCCTTGAACTTTGTTTC  //  ACAACCCAACAGTTTTCTTCTCTCAAAAATG WT Type I
CCTTTCTCTATATAAACCCCTT-TGCCTTGAACTTTGTTTC  //  ACAACCCAACAGTTTTCTTCTCTCAAAAATG mutant allele 1

CCTTTCTCTATATAAACCCCTTTTGCCTTAACTTTGTTTC   //  ACAACCCAACAGTTTTCTTCTCTCAAAAATG WT Type II
CCTTTCTCTATATAAACCCCTTT-------------TTTC   //  ACAACCCAACAGTTTTCTTCTCTCAAAAATG mutant allele 2
```

Line #3

mutant allele 1

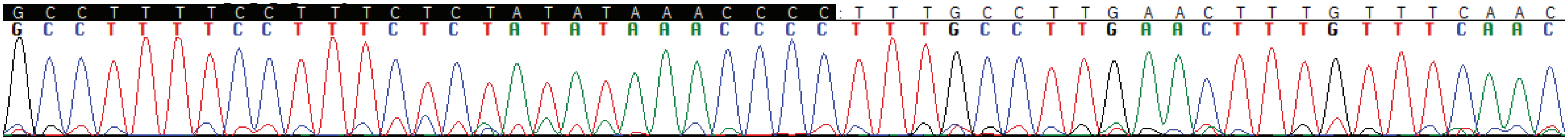

mutant allele 2

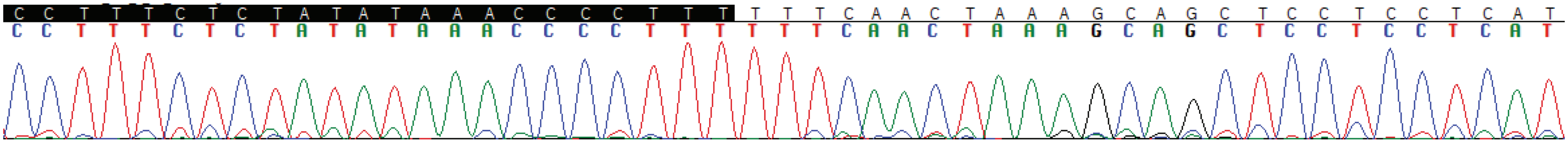

Supplementary Fig. 4

Line #5

```
CCTTTCTCTATATAAACCCTTTTGCCTTGAACTTTGTTTC  //  ACAACCCAACAGTTTTCTTCTCTCAAAAATG  Type I
CCTTTCTCTATATAAACCCTTTT---TTGAACTTTGTTTC  //  ACAACCCAACAGTTTTCTTCTCTCAAAAATG  mutant allele 1

CCTTTCTCTATATAAACCCTTTTGCCTTAACTTTGTTTC   //  ACAACCCAACAGTTTTCTTCTCTCAAAAATGGAATGCAAACACAAAATTAATGTAGCA  Type II
CCTTTCTCTATATAAA---------------------------------------------------------------------------ATTAATGTAGCA  mutant allele 2
```

mutant allele 1

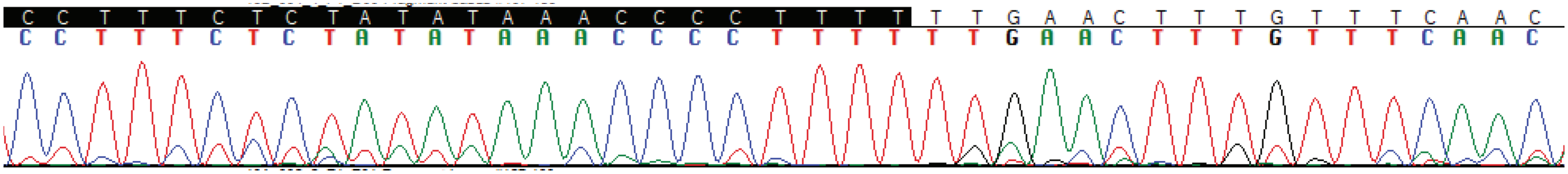

mutant allele 2

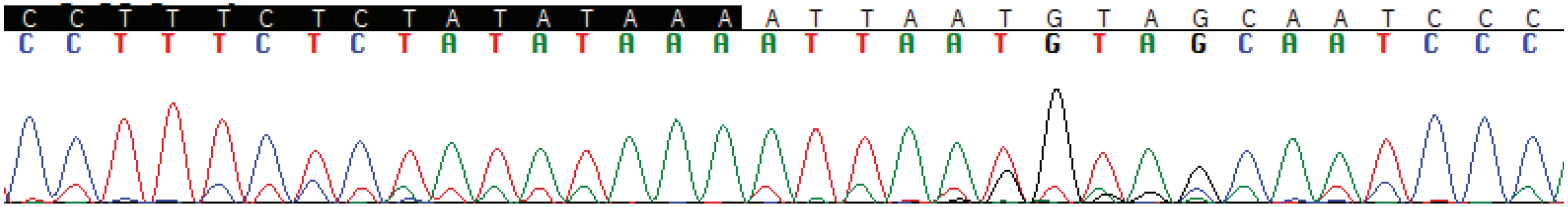

Supplementary Fig. 4

```
CCTTTCTCTATATAAACCCCTTTTGCCTTGAACTTTGTTTC   //  ACAACCCAACAGTTTTCTTCTCTCAAAAATG type I
                        TT
CCTTTCTCTATATAAACCCCTTTT-----GAACTTTGTTTC   //  ACAACCCAACAGTTTTCTTCTCTCAAAAATG  mutant allele 1
CCTTTCTCTATATAAACCCCTTTTGCCTTAACTTTGTTTC    //  ACAACCCAACAGTTTTCTTCTCTCAAAAATG type II
CCTTTCTCTATATAAACCCCTT-TGCCTTAACTTTGTTTC    //  ACAACCCAACAGTTTTCTTCTCTCAAAAATG mutant allele 2
```

Line #6 & 7

mutant allele 1

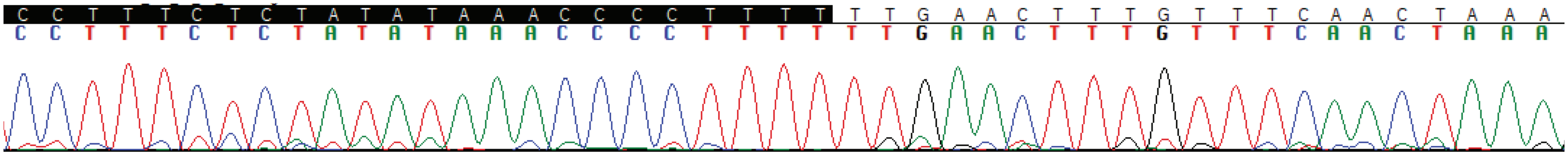

mutant allele 2

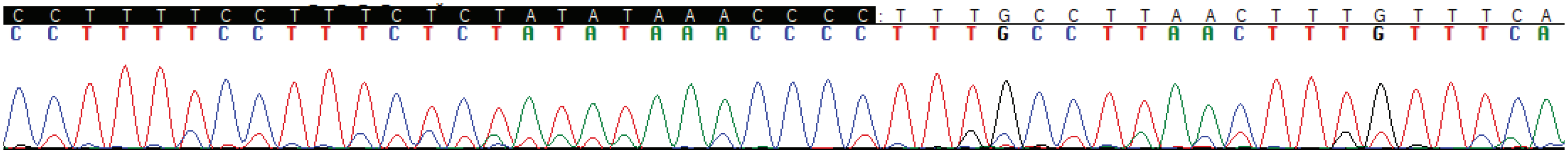

Supplementary Fig. 4

Line #8

```
CCTTTCTCTATATAAACCCCTTTTGCCTTGAACTTTGTTTC  //  ACAACCCAACAGTTTTCTTCTCTCAAAAATG  Type I
CCTTTCTCTATATAAACCCCTTTT---TTGAACTTTGTTTC  //  ACAACCCAACAGTTTTCTTCTCTCAAAAATG  mutant allele 1

CCTTTCTCTATATAAACCCCTTTTGCCTTAACTTTGTTTC   //  ACAACCCAACAGTTTTCTTCTCTCAAAAATG type II
CCTTTCTCTATATAAACCCCTTT----TTAACTTTGTTTC   //  ACAACCCAACAGTTTTCTTCTCTCAAAAATG mutant allele 2
```

mutant allele 1

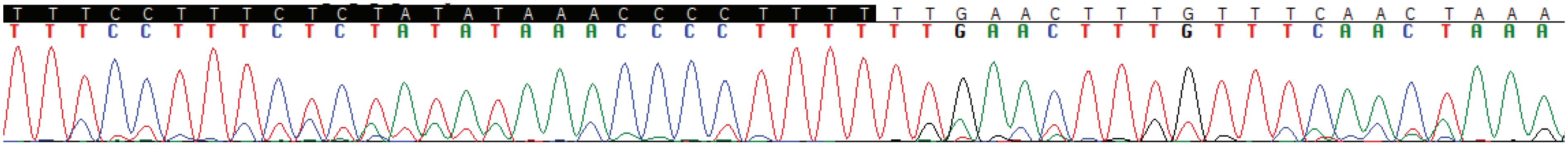

mutant allele 2

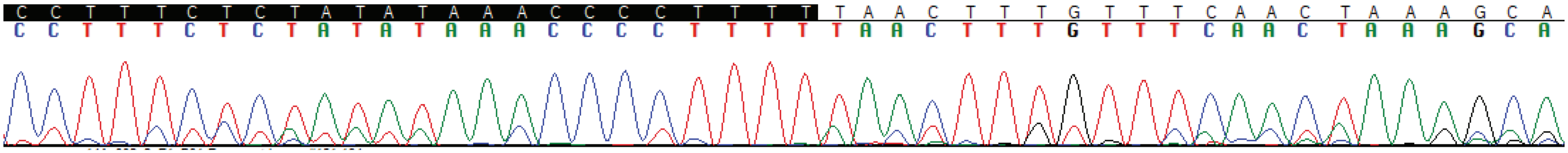

Supplementary Fig. 4

```
CCTTTCTCTATATAAACCCCTTTTGCCTTGAACTTTGTTTC   //  ACAACCCAACAGTTTTCTTCTCTCAAAAATG  type I
CCTTTCTCTATATAAACCCCTTTT-----GAACTTTGTTTC   //  ACAACCCAACAGTTTTCTTCTCTCAAAAATG   mutant allele 1
CCTTTCTCTATATAAACCCCTTTTGCCTTAACTTTGTTTC   //  ACAACCCAACAGTTTTCTTCTCTCAAAAATG type II
                        TT
CCTTTCTCTATATAAACCCCTTTTGCCTTAACTTTGTTTC   //  ACAACCCAACAGTTTTCTTCTCTCAAAAATG mutant allele 2
```

Line #14

mutant allele 1

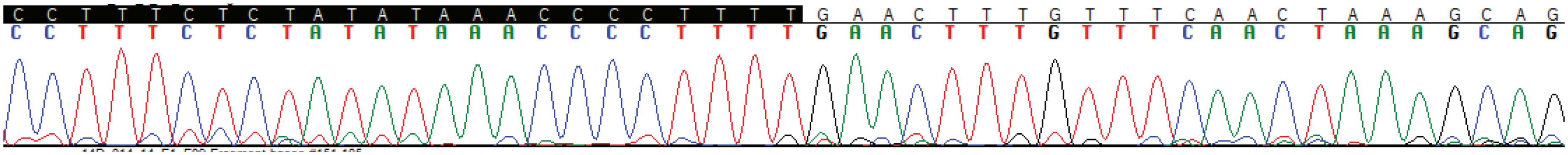

mutant allele 2

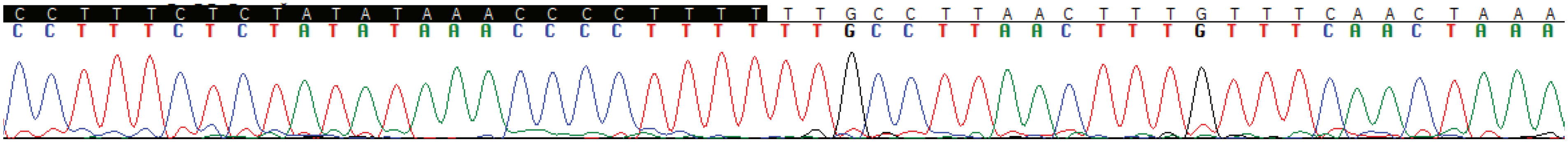

Supplementary Fig. 4

```
CCTTTCTCTATATAAACCCTTTTGCCTTGAACTTTGTTTC  //  ACAACCCAACAGTTTTCTTCTCTCAAAAATG WT Type I (-7 bp)
CCTTTCTCTATATAAACCCTTT-------AACTTTGTTTC  //  ACAACCCAACAGTTTTCTTCTCTCAAAAATG mutant allele 1

CCTTTCTCTATATAAACCCTTTTGCCTTAACTTTGTTTC   //  ACAACCCAACAGTTTTCTTCTCTCAAAAATG WT Type II (+2 bp)
                      TT
CCTTTCTCTATATAAACCCTTTTGCCTTAACTTTGTTTC   //  ACAACCCAACAGTTTTCTTCTCTCAAAAATG mutant allele 2
```

Line #15

mutant allele 1

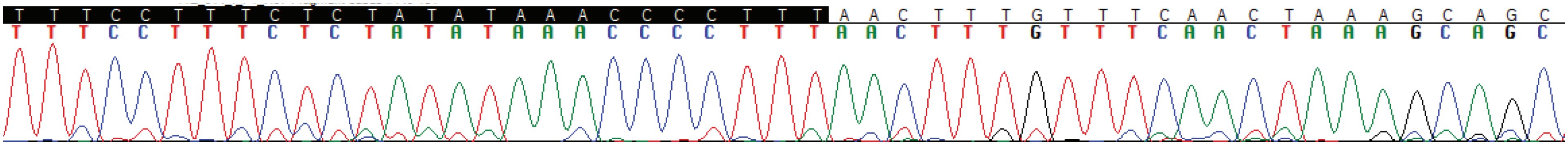

mutant allele 2

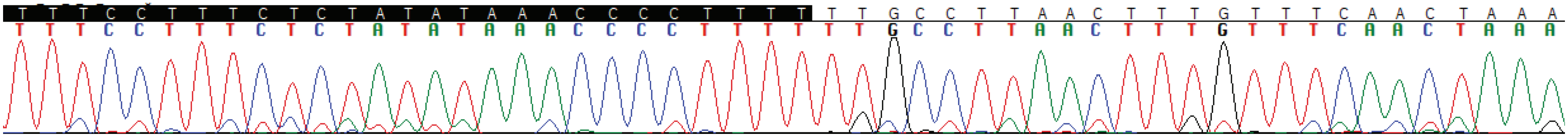

Supplementary Fig. 4

```
CCTTTCTCTATATAAACCCCTTTTGCCTTGAACTTTGTTTC   //  ACAACCCAACAGTTTTCTTCTCTCAAAAATG  type I
CCTTTCTCTATATAAACCCCTTTTG-----AACTTTGTTTC   //  ACAACCCAACAGTTTTCTTCTCTCAAAAATG   mutant allele 1
CCTTTCTCTATATAAACCCCTTT-------AACTTTGTTTC   //  ACAACCCAACAGTTTTCTTCTCTCAAAAATG   mutant allele 2

CCTTTCTCTATATAAACCCCTTTTGCCTTAACTTTGTTTC   //  ACAACCCAACAGTTTTCTTCTCTCAAAAATG  type II
CCTTTCTCTATATAAACCCCTTTT------------------------------------AGTTTTCTTCTCTCAAAAATG   mutant allele 3
```

Line #16

mutant allele 1

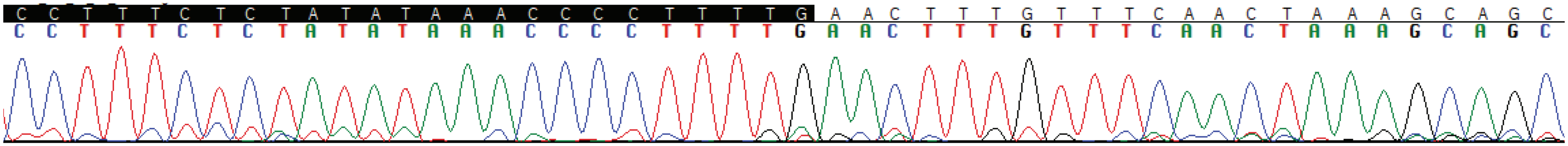

mutant allele 2

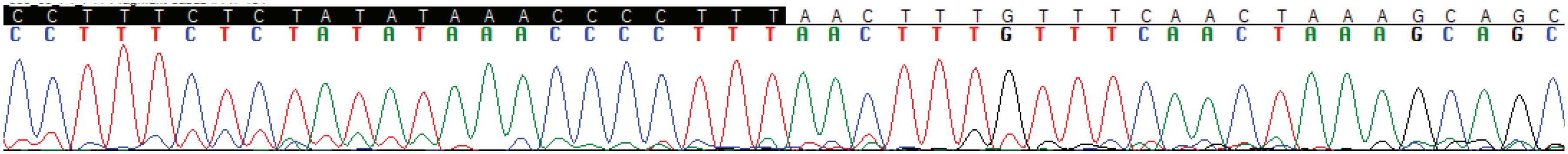

mutant allele 3

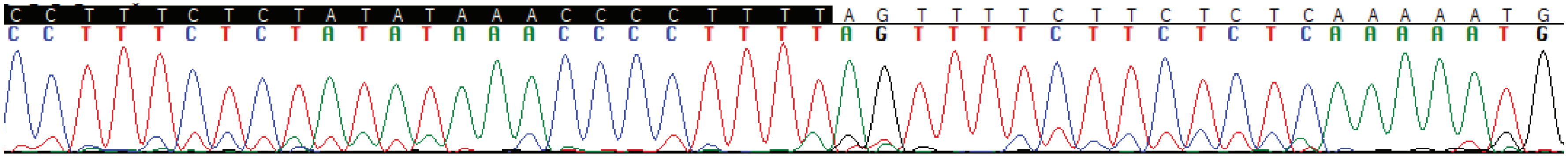

Supplementary Fig. 4

```
CCTTTCTCTATATAAACCCCTTTTGCCTTGAACTTTGTTTC  //  ACAACCCAACAGTTTTCTTCTCTCAAAAATG WT Type I
                          G
CCTTTCTCTATATAAACCCCTTTTGCCTTGAACTTTGTTTC  //  ACAACCCAACAGTTTTCTTCTCTCAAAAATG mutant allele 1

CCTTTCTCTATATAAACCCCTTTTGCCTTAACTTTGTTTC   //  ACAACCCAACAGTTTTCTTCTCTCAAAAATG WT Type II
                         TT
CCTTTCTCTATATAAACCCCTTTTGCCTTAACTTTGTTTC   //  ACAACCCAACAGTTTTCTTCTCTCAAAAATG mutant allele 2
```

Line #11 (heterozygous)

WT allele 1

mutant allele 1

WT allele 2

mutant allele 2

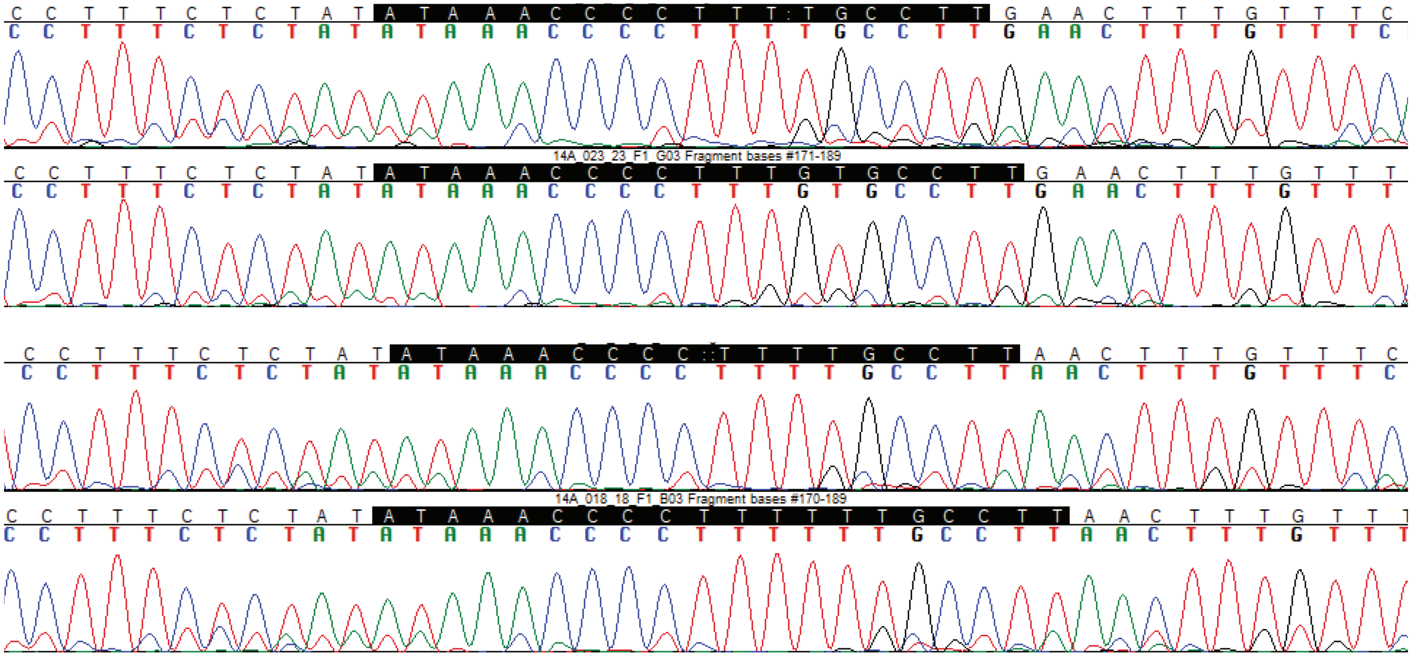

Supplementary Fig. 4 Genotyping of EBE-edited Hamlin sweet orange lines.

Supplementary Table 1 Expression levels of citrus ubiquitin genes at different leaf developmental stages from the RNA-seq data

Genes	Leaf stages			
	V3	V4	V5	V6
Cs6g04450	398.8756	309.2921	514.0988	497.2094
Cs6g04470	57.87721	53.94263	67.12759	60.64882
Cs4g11150	88.14228	75.90725	195.0043	154.7267
Cs4g11160	26.71857	17.95612	28.1004	27.11233
Cs4g11190	496.6204	409.6152	556.3492	715.6756
Cs6g04500	25.27439	20.24717	22.32457	17.32931

Note: V3 and V4: flush expansion, V4 and V5 leaf expansion, V5 and V6: leaf maturation

Supplementary Table 2 Genotyping of EBE mutants

Line	aka	Mutation type	EBE allele type (type G)	EBE allele type (type -)	# of clones sequenced	Survived or died after micro-grafting
#1	Ham1	Biallelic	insert TT (X 19)	delete 24 bp (x7)	26	Survived
#2	Ham2	Biallelic	Insert TA (X9)	Insert TT (X18)	27	Survived
#3	Ham3	Biallelic	delete T (X 15)	delete 14 bp (x 14)	29	Survived
#4	Ham4	Biallelic	insert AT (X 17)	insert TT (X 15)	32	Survived
#5	Ham5	Biallelic	delete GCC (X 10)	124 bp large deletion (x 5)	15	Survived
#6	Ham6	Biallelic	delete GCC (X 10)	delete T (x 6)	16	Died
#7	Ham7	Biallelic	delete GCC (x 15)	delete T (x 19)	34	Died
#8	Ham8	Biallelic	delete GCC (x 11)	delete TGCC (x 10)	21	Died
#9	Ham9	heterozygous				Died
#10	Ham10	heterozygous				Survived
#11	Ham11	heterozygous				Survived
#12	Ham12	na	na	na		na
#13	Ham13	na	na	na		na
#14	Ham14	Biallelic	delete 5 bp (x 13)	insert TT (X 15)	28	Died
#15	Ham15	Biallelic	delete TGCCTTG (x 11)	insert TT (X 7)	18	Survived
#16	Ham16	Null	delete 5 bp, 7 bp (x 10)	80 bp large deletion (x 6)	16	Died

Supplementary Table 3 List of primers used in this study

Primer name	Sequence (from 5' to 3')	Purposes
CsUbi-F1	CAACAAAGGGTAATATCCGGAGGTTTTATCAATAAATCTCCATGATAC	Amplification of CsUbi promoter
CsUbi-R1	AGTACTTCTTATCCATGTCGACCTGTTGGACCAAAGAAACACCATAT	Amplification of CsUbi promoter
CmYLCV-F2	CAACAAAGGGTAATATCCGGATGGCAGACATACTGTCCCACAAA	Amplification of CmYLCV promoter
CmYLCV-R2	AGTACTTCTTATCCATGTCGACAAGCTTAGCTCTTACCTGTTTTCG	Amplification of CmYLCV promoter
LOBPro3-F1	AtGGTCTCGtgcaAAACAAAGTTCAAGGCAAAAGTTTAAGAGCTATGCTGGAAACA	For CRISPR construct to edit CsLOB1 EBE region
LOBPro3-R1	atGGTCTCCTCTTCTCTCAAATGCACCAGCCGGGAATCGAAC	For CRISPR construct to edit CsLOB1 EBE region
LOBPro3-F2	AtGGTCTCGaagaAAACTGTTGTTTAAGAGCTATGCTGGAAACAG	For CRISPR construct to edit CsLOB1 EBE region
LOBPro3-R2	ctaGGTCTCCAAACTTTTGCCTTAACTTTGTTTCTGCACCAGCCGGGAATCGAAC	For CRISPR construct to edit CsLOB1 EBE region
LOBpro-F1	CGATAAAATTCACCTCCATGTAATT	For EBE mutant genotyping
LOBpro-R1	GAAGCGTGGAGAAGATTGAGAAG	For EBE mutant genotyping
GAPDH-F1	GAAAGGTCTTGCCTGCTTTG	For qRT-PCR normalization
GAPDH-R1	TCCTTCTCCAGCCTCACTGT	For qRT-PCR normalization
QLOB1-F1	TCCACCAACCGAACCATACA	For LOB1 qRT-PCR
QLOB1-R1	GGCACTTGCTTCATAGACCAT	For LOB1 qRT-PCR
QLOB2-F1	CCACAAAATTTCATCATTGTTCAT	For LOB2 qRT-PCR
QLOB2-R1	GCTCCTTCTGTCCCTATAGTTTGA	For LOB2 qRT-PCR
NtPDS1	AtGGTCTCGtgcaGAGATTGTTATTGCTGGTGCGTTTAAGAGCTATGCTGGAAACA	For CRISPR construct to edit tobacco NtPDS region
NtPDS2	taggtctcgAAACTCATCATCTTTCCATGCAGCTGCACCAGCCGGGAATCGAACCC	For CRISPR construct to edit tobacco NtPDS region
Cas9gt-F1	AACTAACTCTGTGGGATGGGCTGT	For detection of Cas9 in transformants
Cas9gt-R1	TCTCGTGGTATGCCACCTCATCAA	For detection of Cas9 in transformants

For off-targets:

Off1-R1	TTCCAGAGACATCACAGTTGTAG
Off2-F1	CTGCTAATACTCTCTGTTTCAGC
Off2-R1	AATTTGTAGCGCTGATTCCAGAG
Off3-F1	CATGTATTCTAGTTCGATTGAAGAC
Off3-R1	GCTTATTAAACAAAACACTAGCACG
Off4-F1	ATGGAAATAGACAGCGCCAAGAC
Off4-R1	TAAATACGAGGTACAGCAGAGGC
Off5-F1	TTGGGAAAGATATGTTTACTATGGA
Off5-R1	ACAGAATTAACAACTTGTCTTCCG
Off6-F1	TTTCCATTGCTGCACTCAATGTG
Off6-R1	TGATGCAGTTAAGGAGAGTTCTG
Off7-F1	AAGTATAAACTAAGGTAATATTCAACC
Off7-R1	GTCATAGGTTGAAGCCTTACTATG
Off8-F1	ACACAAGAATCAAGTATAAACTAAGG
Off8-R1	AGGCTCCACTTCATGAACCTGC
Off9-F1	CTTTCTAAGAACCATATTCATTGTC
Off9-R1	CCTTACTATGAAAACTTTCAGACAC
Off10-F1	GTCATAGGTTGAAGCCTTACTATG
Off10-R1	AAGTATAAACTAAGGTAATATTCAACC
Off11-F1	TCTGGCGAAATTTGCCATAGACT
Off11-R1	TTTATATCCTTTCTACAAAGGTTCG
Off12-F1	CGATCCTTGACTATCCTTGGTC
Off12-R1	AGCAAACAATGCTATAAACATAAGG

Off13-F1	CTCGACCGTAAAAAGACGAGAG
Off13-R1	AGCAAACAATGCTATAAACATAAGG
Off14-F1	TTACCTCCCTTAAAGGTAAATTAAG
Off14-R1	ATAACGGAGATTTTTACCCTTTATG
Off15-F1	CTCCCTTAAAGGTAAATTAAGTACA
Off15-R1	AAAAAAATACCAATTTTACCCTTTATG
Off16-F1	TCCATTTCAAGTTTCCACAGTCAG
Off16-R1	TAACACCACCACAGAACCTGAAC
Off17-F1	TCATCATCAACATTGGTGTGCTTG
Off17-R1	AGAAAACCGAATAAATTAGTGGTTTC
Off18-F1	AACAATTATTTACTTGAGCTGCAAGT
Off18-R1	TTTCCGGAGAGGTTACAACAAGC
Off19-F1	AATTCGTGTTCTACAATGGGTCAG
Off19-R1	ATGAACTTCGTGCCTTAATTCTCG
Off20-F1	ATCGGGTTCAGATTCAGATCCGA
Off20-R1	ACTGATTATGTAAGAAACAAGCAATC

Supplementary Table 4 Summary for deep sequencing of amplicon derived from samples in Supplementary Fig. 1A (incubated at 30°C)

**Genotype**	**Sample 1**	**Sample 2**	**Sample 3**
WT genotype	81.22%	81.88%	83.08%
86 bp deletion	8.56%	7.41%	6.44%
88 bp deletion	2.81%	2.66%	2.21%
2 bp deletion	0.61%	0.48%	0.44%
4 bp deletion	0.19%	0.14%	0.12%
1bp insertion	0.19%	0.19%	0.15%
substitute (A->G)	3.06%	3.57%	3.42%
substitute (C->T)	2.85%	3.31%	3.51%
substitute (A->C)	0.50%	0.37%	0.63%

Supplementary Table 5 Off target analyses of the 6 canker-resistant Hamlin edited lines

#	Off-target site	Locus	Gene	Region	Off-target edits					
					Line 1	Line 2	Line 3	Line 4	Line 5	Line 15
1	AAAAAAAGAAAAAGGCAAAAGGG	chrUn:-41544413		Intergenic	No	No	No	No	No	No
2	AAAAAAAGAAAAAGGCAAAAGGG	chrUn:-41545551		Intergenic	No	No	No	No	No	No
3	CAACATAGAACAAGGCAAAATGG	chrUn:+42303008		Intergenic	No	No	No	No	No	No
4	AAAAAAAATTAAGGGCAAAACGG	chrUn:+62946829		Intergenic	No	No	No	No	No	No
5	AAACAAATTACAAAGAAAAATGG	chrUn:-18572431	orange1.1t01132	utr	No	No	No	No	No	No
6	AAAGAGAAATCAAGGCAAAATGG	chr5:-7443270	Cs5g10590	CDS	No	No	No	No	No	No
7	AAACAAAATTCAACACAAAGTGG	chrUn:+20408113		Intergenic	No	No	No	No	No	No
8	AAACAAAATTCAACACAAAGTGG	chr1:+21980682		Intergenic	No	No	No	No	No	No
9	AAACAAAATTCAACACAAAGTGG	chrUn:+18619356		Intergenic	No	No	No	No	No	No
10	AAACAAAATTCAACACAAAGTGG	chr3:-9334994		Intergenic	No	No	No	No	No	No
11	TTTGGAAGAAGAAAAATTTTAGG	chr2:+20170295		Intergenic	No	No	No	No	No	No
12	TTTAGGAGAAGAAAAATTTTTGG	chrUn:-72068007		Intergenic	No	No	No	No	No	No
13	TTTAGGAGAAGAAAAATTTTTGG	chrUn:-72096901		Intergenic	No	No	No	No	No	No
14	TTTGAAAAAAAAAAATTGTTGGG	chr8:+12348262		Intergenic	No	No	No	No	No	No
15	TTTGAAAAAAAAAAATTGTTGGG	chr8:+12359484		Intergenic	No	No	No	No	No	No
16	GATGAGAGCAGAAAACTTTTTGG	chrUn:+28429842		Intergenic	No	No	No	No	No	No
17	TTTGAGAAGAGAAAGCTTTTAGG	chr2:-12040216	Cs2g15250	intron	No	No	No	No	No	No
18	TATGAGAAAAAAAAATTGTTAGG	chr8:+3538420	Cs8g06040	intron	No	No	No	No	No	No
19	TTGGAGGGAAAGAAACTGTTTGG	chr1:+19083773		Intergenic	No	No	No	No	No	No
20	TTTGAGGAAAGTAAAATGTTTGG	chr2:+7402647	Cs2g10040	utr	No	No	No	No	No	No

Supplementary Data 1

35S enhancer-CmYLCV promoter:

Yellow, 35S enhancer; **Black, CmYLCV Promoter**

GCATGCGGCGCGCCGATCGGCGCGCCAGATTTGCCTTTTCAATTTCAGAAAGAATGCTAACCCA
CAGATGGTTAGAGAGGCTTACGCAGCAGGTATCATCAAGACGATCTACCCGAGCAATAATCTCC
AGGAAATCAAATACCTTCCCAAGAAGGTTAAAGATGCAGTCAAAAGATTCAGGACTAACTGCAT
CAAGAACACAGAGAAAGATATATTTCTCAAGATCAGAAGTACTATTCCAGTATGGACGATTCAA
GGCTTGCTTCACAAACCAAGGCAAGTAATAGAGATTGGAGTCTCTAAAAAGGTAGTTCCCACTG
AATCAAAGGCCATGGAGTCAAAGATTCAAATAGAGGACCTAACAGAACTCGCCGTAAAGACTGG
CGAACAGTTCATACAGAGTCTCTTACGACTCAATGACAAGAAGAAAATCTTCGTCAACATGGTG
GAGCACGACACACTTGTCTACTCCAAAAATATCAAAGATACAGTCTCAGAAGACCAAAGGGCAA
TTGAGACTTTTCAACAAAGGGTAATATCCGGA**TGGCAGACATACTGTCCCACAAATGAAGATGG**
AATCTGTAAAAGAAAACGCGTGAAATAATGCGTCTGACAAAGGTTAGGTCGGCTGCCTTTAATC
AATACCAAAGTGGTCCCTACCACGATGGAAAAACTGTGCAGTCGGTTTGGCTTTTTCTGACGAA
CAAATAAGATTCGTGGCCGACAGGTGGGGGTCCACCATGTGAAGGCATCTTCAGACTCCAATAA
TGGAGCAATGACGTAAGGGCTTACGAAATAAGTAAGGGTAGTTTGGGAAATGTCCACTCACCCG
TCAGTCTATAAATACTTAGCCCCTCCCTCATTGTTAAGGGAGCAAAATCTCAGAGAGATAGTCC
TAGAGAGAGAAAGAGAGCAAGTAGCCTAGAAGTAGTCAAGGCGGCGAAGTATTCAGGCACGTGG
CCAGGAAGAAGAAAAGCCAAGACGACGAAAACAGGTAAGAGCTAAGCTT

35S enhancer-CsUbi promoter:

Yellow, 35S enhancer; **Black, CsUbi Promoter**

GCATGCGGCGCGCCGATCGGCGCGCCAGATTTGCCTTTTCAATTTCAGAAAGAATGCTAACCCA
CAGATGGTTAGAGAGGCTTACGCAGCAGGTATCATCAAGACGATCTACCCGAGCAATAATCTCC
AGGAAATCAAATACCTTCCCAAGAAGGTTAAAGATGCAGTCAAAAGATTCAGGACTAACTGCAT
CAAGAACACAGAGAAAGATATATTTCTCAAGATCAGAAGTACTATTCCAGTATGGACGATTCAA
GGCTTGCTTCACAAACCAAGGCAAGTAATAGAGATTGGAGTCTCTAAAAAGGTAGTTCCCACTG
AATCAAAGGCCATGGAGTCAAAGATTCAAATAGAGGACCTAACAGAACTCGCCGTAAAGACTGG
CGAACAGTTCATACAGAGTCTCTTACGACTCAATGACAAGAAGAAAATCTTCGTCAACATGGTG
GAGCACGACACACTTGTCTACTCCAAAAATATCAAAGATACAGTCTCAGAAGACCAAAGGGCAA
TTGAGACTTTTCAACAAAGGGTAATATCCGGA**GGTTTTATCAATAAATCTCCATGATACTTTTA**
TTGAAAATTGTCATAAATTATCAACACGGATTATCAACCTAATTGAGAGTTTTCAATTTTATAT
TTTTTTAGTGATTGTAAAATTTCCCTCTTATTCCTAAATCACTATATTTATTTATGATTAATTC

AATTACGCTACTAGAGTGATTCTTATAATTTTGGTTGAGTAGTTTTCACTTTCATCCAGCTCAA
AAACTTAAAATTACAAATTGAAAAAAAAATTTGGATTTTATTATCAGAAATGCTGTTATAGGAA
TCTTTAAATTTTCGCAGATGTTCTTCTCAGAAAAACTTATTTAACATTTTTTTTAAGATAACTT
TAAATCGTTAAAAATAATCCACAAATATACAAATAGAAAATTAGAATGATGAGCTACCGTGTGA
GGTGTGGCACACTCGTATCGAAGCGCGTGACTTGAATGCTAAACAATATCCTAAAATCTGAAAG
ATATCCGCCTCTTCCGATCAGCCAACGGTTTTGCGAAACTACTATTGGCGTGTGGCCAGGAAA
GGGACACCGAATGTTAAAGACGTGGCATCAATGTGGTGGATGGAATTTGGGCAATCTCGTCATT
TTAATTGTCAATCACGACTATAAAATGGAGGACCTCGAACCTCGGCTCCCCACCGTTTCTCAGA
TTATCTTCACCTTTAATTCGATAAAAGGCCTCCTTCTGTTCTCTCTGTCAAGGTAATAATGATT
ATAATTCATCTCTTTAGCATTTTGGTTTTTTTTTAAAATTAATATTTATTTTACCGATTATTCT
AGCTGCTATTATGTCGTGCTTTGTCGATTAATTCGTAGATTTTTTTTTTTTAGTTATTATTGTTT
ATCTTGATATGGTGATATGCTATTTATAAGCTTTTCATTAGCTGTAAACTTTTGTAGGCGATCG
CTTATTTGTTGTTTGAGGTTATAATTATGGCGATTTAACACTCAAATCAGCCTCGTTGATTCTA
TCTAGGGTTTTGATTTAATGGTGTTATAGTGCTCTTTCGGAGATAATTTTGAATTTTTTGGTGT
TAATTTTAGGCGTATCTTGTAATTCTATGAGACTTGTGAAAAAAAAATAATTAACAAAGAAAAA
GGTATGCCGAAATCTTTACTTATGATTGATCGTTCTTGAAAATCTCTTTTCTATTTTTTGATCT
ATCTAGAGATCTACAACTTTTCTAAATTTTCTGTTAGTTTTTCCTTGAGAGACCTGCCCGAAAG
TGTCTTTGAATTATGTAGTGACTACGTCTAACACAACTCTGTGTCGTTGGTTATGTTAATATTG
TTTGTTTGGTTTGATTGTTTTGATTGTTTTGTATGGATTTATTCTGATGTGGTGTTTCTTTGGT
CCAACAGGTCGAC

CsU6 promoter (also known as CsU6-2 promoter):

CGCTCAGGAGCCGGTTGAATTTGATTGTTGTTTGATGTTTAGGTATGCTTACAATTTTTTACTA
AATAAGGTAATAATTGTTCCTTTTATTTTATTCATTTAGCTTGGTACTTGTCGTAATCTATCTC
CGTCAATGTCAGCTTCTTCTGTGTCGGAAGAAGCTGAAAAATGTTGTTGAAGGCCCTTTCATTT
ATTATTATCATTATTATGTTTAATTTTGTTCGGCAAATGTGTACTAATTTGGTTCGAATCGTTC
GATCTCATAATACAAACATGTAATGAGTTATTTATGACGTCATGGATGCGGTTAGTTTGCGTGG
TCTGAAATTTCAACCAAATTATTCAATCATGGTGGTGGCCGGCTGGTGCCTATCCCTTGATTGA
AATATGCAATTAAAATTCTCGTAATAATGTTGATTTGTTCTATATACTTGAATTGATGGTATTA
ATTATTGTTATAAAAGCACTAACTTGTTTGAGAAAGGGGATAACTAAAAGGTAATAATAATAAT
AATAAAATAAAGAAAAGGCCCAACACATGGGCGCCCTATCTGTTGGGCCAAATGCCATAAGAGG
ATCCAGCTAGCCCGTTGAAGAAAATCCCACATCGAAAGAAAAAACTGAATAAACATGGTCTATA
TATACAAGGACTCCCAGGTTGGTTG

CsU6 3’ region:

3’ region regulatory sequence of citrus CsU6 gene (downstream of PolyT)

CCCCTGTTTTTTCCTAATTAGATTTCTTTCGGAGCTGTTCGAAGAACATGTTTTTGCCGTCTGA
CTCGTTTGTTATTGCCGTTCTAGTTAGTTTCAGCTATTGGTTTTCGGTTTCTTTCTGTTTTGAA

GGGTAATAGTTTGGAAAATATAATACAAATTAATTTAGGATTAAAAAGTAATAAAACAATATTA
TCTAGA
